# Search for doubly charged scalar bosons decaying into same-sign *W* boson pairs with the ATLAS detector

**DOI:** 10.1140/epjc/s10052-018-6500-y

**Published:** 2019-01-23

**Authors:** M. Aaboud, G. Aad, B. Abbott, O. Abdinov, B. Abeloos, D. K. Abhayasinghe, S. H. Abidi, O. S. AbouZeid, N. L. Abraham, H. Abramowicz, H. Abreu, Y. Abulaiti, B. S. Acharya, S. Adachi, L. Adamczyk, J. Adelman, M. Adersberger, A. Adiguzel, T. Adye, A. A. Affolder, Y. Afik, C. Agheorghiesei, J. A. Aguilar-Saavedra, F. Ahmadov, G. Aielli, S. Akatsuka, T. P. A. Åkesson, E. Akilli, A. V. Akimov, G. L. Alberghi, J. Albert, P. Albicocco, M. J. Alconada Verzini, S. Alderweireldt, M. Aleksa, I. N. Aleksandrov, C. Alexa, T. Alexopoulos, M. Alhroob, B. Ali, G. Alimonti, J. Alison, S. P. Alkire, C. Allaire, B. M. M. Allbrooke, B. W. Allen, P. P. Allport, A. Aloisio, A. Alonso, F. Alonso, C. Alpigiani, A. A. Alshehri, M. I. Alstaty, B. Alvarez Gonzalez, D. Álvarez Piqueras, M. G. Alviggi, B. T. Amadio, Y. Amaral Coutinho, L. Ambroz, C. Amelung, D. Amidei, S. P. Amor Dos Santos, S. Amoroso, C. S. Amrouche, C. Anastopoulos, L. S. Ancu, N. Andari, T. Andeen, C. F. Anders, J. K. Anders, K. J. Anderson, A. Andreazza, V. Andrei, C. R. Anelli, S. Angelidakis, I. Angelozzi, A. Angerami, A. V. Anisenkov, A. Annovi, C. Antel, M. T. Anthony, M. Antonelli, D. J. A. Antrim, F. Anulli, M. Aoki, J. A. Aparisi Pozo, L. Aperio Bella, G. Arabidze, J. P. Araque, V. Araujo Ferraz, R. Araujo Pereira, A. T. H. Arce, R. E. Ardell, F. A. Arduh, J-F. Arguin, S. Argyropoulos, A. J. Armbruster, L. J. Armitage, A Armstrong, O. Arnaez, H. Arnold, M. Arratia, O. Arslan, A. Artamonov, G. Artoni, S. Artz, S. Asai, N. Asbah, A. Ashkenazi, E. M. Asimakopoulou, L. Asquith, K. Assamagan, R. Astalos, R. J. Atkin, M. Atkinson, N. B. Atlay, K. Augsten, G. Avolio, R. Avramidou, M. K. Ayoub, G. Azuelos, A. E. Baas, M. J. Baca, H. Bachacou, K. Bachas, M. Backes, P. Bagnaia, M. Bahmani, H. Bahrasemani, A. J. Bailey, J. T. Baines, M. Bajic, C. Bakalis, O. K. Baker, P. J. Bakker, D. Bakshi Gupta, E. M. Baldin, P. Balek, F. Balli, W. K. Balunas, J. Balz, E. Banas, A. Bandyopadhyay, S. Banerjee, A. A. E. Bannoura, L. Barak, W. M. Barbe, E. L. Barberio, D. Barberis, M. Barbero, T. Barillari, M-S. Barisits, J. Barkeloo, T. Barklow, N. Barlow, R. Barnea, S. L. Barnes, B. M. Barnett, R. M. Barnett, Z. Barnovska-Blenessy, A. Baroncelli, G. Barone, A. J. Barr, L. Barranco Navarro, F. Barreiro, J. Barreiro Guimarães da Costa, R. Bartoldus, A. E. Barton, P. Bartos, A. Basalaev, A. Bassalat, R. L. Bates, S. J. Batista, S. Batlamous, J. R. Batley, M. Battaglia, M. Bauce, F. Bauer, K. T. Bauer, H. S. Bawa, J. B. Beacham, M. D. Beattie, T. Beau, P. H. Beauchemin, P. Bechtle, H. C. Beck, H. P. Beck, K. Becker, M. Becker, C. Becot, A. Beddall, A. J. Beddall, V. A. Bednyakov, M. Bedognetti, C. P. Bee, T. A. Beermann, M. Begalli, M. Begel, A. Behera, J. K. Behr, A. S. Bell, G. Bella, L. Bellagamba, A. Bellerive, M. Bellomo, P. Bellos, K. Belotskiy, N. L. Belyaev, O. Benary, D. Benchekroun, M. Bender, N. Benekos, Y. Benhammou, E. Benhar Noccioli, J. Benitez, D. P. Benjamin, M. Benoit, J. R. Bensinger, S. Bentvelsen, L. Beresford, M. Beretta, D. Berge, E. Bergeaas Kuutmann, N. Berger, L. J. Bergsten, J. Beringer, S. Berlendis, N. R. Bernard, G. Bernardi, C. Bernius, F. U. Bernlochner, T. Berry, P. Berta, C. Bertella, G. Bertoli, I. A. Bertram, G. J. Besjes, O. Bessidskaia Bylund, M. Bessner, N. Besson, A. Bethani, S. Bethke, A. Betti, A. J. Bevan, J. Beyer, R. M. Bianchi, O. Biebel, D. Biedermann, R. Bielski, K. Bierwagen, N. V. Biesuz, M. Biglietti, T. R. V. Billoud, M. Bindi, A. Bingul, C. Bini, S. Biondi, M. Birman, T. Bisanz, J. P. Biswal, C. Bittrich, D. M. Bjergaard, J. E. Black, K. M. Black, T. Blazek, I. Bloch, C. Blocker, A. Blue, U. Blumenschein, Dr. Blunier, G. J. Bobbink, V. S. Bobrovnikov, S. S. Bocchetta, A. Bocci, D. Boerner, D. Bogavac, A. G. Bogdanchikov, C. Bohm, V. Boisvert, P. Bokan, T. Bold, A. S. Boldyrev, A. E. Bolz, M. Bomben, M. Bona, J. S. Bonilla, M. Boonekamp, A. Borisov, G. Borissov, J. Bortfeldt, D. Bortoletto, V. Bortolotto, D. Boscherini, M. Bosman, J. D. Bossio Sola, K. Bouaouda, J. Boudreau, E. V. Bouhova-Thacker, D. Boumediene, C. Bourdarios, S. K. Boutle, A. Boveia, J. Boyd, I. R. Boyko, A. J. Bozson, J. Bracinik, N. Brahimi, A. Brandt, G. Brandt, O. Brandt, F. Braren, U. Bratzler, B. Brau, J. E. Brau, W. D. Breaden Madden, K. Brendlinger, A. J. Brennan, L. Brenner, R. Brenner, S. Bressler, B. Brickwedde, D. L. Briglin, D. Britton, D. Britzger, I. Brock, R. Brock, G. Brooijmans, T. Brooks, W. K. Brooks, E. Brost, J. H Broughton, P. A. Bruckman de Renstrom, D. Bruncko, A. Bruni, G. Bruni, L. S. Bruni, S. Bruno, B. H. Brunt, M. Bruschi, N. Bruscino, P. Bryant, L. Bryngemark, T. Buanes, Q. Buat, P. Buchholz, A. G. Buckley, I. A. Budagov, F. Buehrer, M. K. Bugge, O. Bulekov, D. Bullock, T. J. Burch, S. Burdin, C. D. Burgard, A. M. Burger, B. Burghgrave, K. Burka, S. Burke, I. Burmeister, J. T. P. Burr, D. Büscher, V. Büscher, E. Buschmann, P. Bussey, J. M. Butler, C. M. Buttar, J. M. Butterworth, P. Butti, W. Buttinger, A. Buzatu, A. R. Buzykaev, G. Cabras, S. Cabrera Urbán, D. Caforio, H. Cai, V. M. M. Cairo, O. Cakir, N. Calace, P. Calafiura, A. Calandri, G. Calderini, P. Calfayan, G. Callea, L. P. Caloba, S. Calvente Lopez, D. Calvet, S. Calvet, T. P. Calvet, M. Calvetti, R. Camacho Toro, S. Camarda, P. Camarri, D. Cameron, R. Caminal Armadans, C. Camincher, S. Campana, M. Campanelli, A. Camplani, A. Campoverde, V. Canale, M. Cano Bret, J. Cantero, T. Cao, Y. Cao, M. D. M. Capeans Garrido, I. Caprini, M. Caprini, M. Capua, R. M. Carbone, R. Cardarelli, F. C. Cardillo, I. Carli, T. Carli, G. Carlino, B. T. Carlson, L. Carminati, R. M. D. Carney, S. Caron, E. Carquin, S. Carrá, G. D. Carrillo-Montoya, D. Casadei, M. P. Casado, A. F. Casha, M. Casolino, D. W. Casper, R. Castelijn, F. L. Castillo, V. Castillo Gimenez, N. F. Castro, A. Catinaccio, J. R. Catmore, A. Cattai, J. Caudron, V. Cavaliere, E. Cavallaro, D. Cavalli, M. Cavalli-Sforza, V. Cavasinni, E. Celebi, F. Ceradini, L. Cerda Alberich, A. S. Cerqueira, A. Cerri, L. Cerrito, F. Cerutti, A. Cervelli, S. A. Cetin, A. Chafaq, D Chakraborty, S. K. Chan, W. S. Chan, Y. L. Chan, J. D. Chapman, D. G. Charlton, C. C. Chau, C. A. Chavez Barajas, S. Che, A. Chegwidden, S. Chekanov, S. V. Chekulaev, G. A. Chelkov, M. A. Chelstowska, C. Chen, C. H. Chen, H. Chen, J. Chen, J. Chen, S. Chen, S. J. Chen, X. Chen, Y. Chen, Y-H. Chen, H. C. Cheng, H. J. Cheng, A. Cheplakov, E. Cheremushkina, R. Cherkaoui El Moursli, E. Cheu, K. Cheung, L. Chevalier, V. Chiarella, G. Chiarelli, G. Chiodini, A. S. Chisholm, A. Chitan, I. Chiu, Y. H. Chiu, M. V. Chizhov, K. Choi, A. R. Chomont, S. Chouridou, Y. S. Chow, V. Christodoulou, M. C. Chu, J. Chudoba, A. J. Chuinard, J. J. Chwastowski, L. Chytka, D. Cinca, V. Cindro, I. A. Cioară, A. Ciocio, F. Cirotto, Z. H. Citron, M. Citterio, A. Clark, M. R. Clark, P. J. Clark, C. Clement, Y. Coadou, M. Cobal, A. Coccaro, J. Cochran, A. E. C. Coimbra, L. Colasurdo, B. Cole, A. P. Colijn, J. Collot, P. Conde Muiño, E. Coniavitis, S. H. Connell, I. A. Connelly, S. Constantinescu, F. Conventi, A. M. Cooper-Sarkar, F. Cormier, K. J. R. Cormier, M. Corradi, E. E. Corrigan, F. Corriveau, A. Cortes-Gonzalez, M. J. Costa, D. Costanzo, G. Cottin, G. Cowan, B. E. Cox, J. Crane, K. Cranmer, S. J. Crawley, R. A. Creager, G. Cree, S. Crépé-Renaudin, F. Crescioli, M. Cristinziani, V. Croft, G. Crosetti, A. Cueto, T. Cuhadar Donszelmann, A. R. Cukierman, J. Cúth, S. Czekierda, P. Czodrowski, M. J. Da Cunha Sargedas De Sousa, C. Da Via, W. Dabrowski, T. Dado, S. Dahbi, T. Dai, F. Dallaire, C. Dallapiccola, M. Dam, G. D’amen, J. Damp, J. R. Dandoy, M. F. Daneri, N. P. Dang, N. D Dann, M. Danninger, V. Dao, G. Darbo, S. Darmora, O. Dartsi, A. Dattagupta, T. Daubney, S. D’Auria, W. Davey, C. David, T. Davidek, D. R. Davis, E. Dawe, I. Dawson, K. De, R. De Asmundis, A. De Benedetti, M. De Beurs, S. De Castro, S. De Cecco, N. De Groot, P. de Jong, H. De la Torre, F. De Lorenzi, A. De Maria, D. De Pedis, A. De Salvo, U. De Sanctis, A. De Santo, K. De Vasconcelos Corga, J. B. De Vivie De Regie, C. Debenedetti, D. V. Dedovich, N. Dehghanian, M. Del Gaudio, J. Del Peso, Y. Delabat Diaz, D. Delgove, F. Deliot, C. M. Delitzsch, M. Della Pietra, D. Della Volpe, A. Dell’Acqua, L. Dell’Asta, M. Delmastro, C. Delporte, P. A. Delsart, D. A. DeMarco, S. Demers, M. Demichev, S. P. Denisov, D. Denysiuk, L. D’Eramo, D. Derendarz, J. E. Derkaoui, F. Derue, P. Dervan, K. Desch, C. Deterre, K. Dette, M. R. Devesa, P. O. Deviveiros, A. Dewhurst, S. Dhaliwal, F. A. Di Bello, A. Di Ciaccio, L. Di Ciaccio, W. K. Di Clemente, C. Di Donato, A. Di Girolamo, B. Di Micco, R. Di Nardo, K. F. Di Petrillo, A. Di Simone, R. Di Sipio, D. Di Valentino, C. Diaconu, M. Diamond, F. A. Dias, T. Dias Do Vale, M. A. Diaz, J. Dickinson, E. B. Diehl, J. Dietrich, S. Díez Cornell, A. Dimitrievska, J. Dingfelder, F. Dittus, F. Djama, T. Djobava, J. I. Djuvsland, M. A. B. Do Vale, M. Dobre, D. Dodsworth, C. Doglioni, J. Dolejsi, Z. Dolezal, M. Donadelli, J. Donini, A. D’onofrio, M. D’Onofrio, J. Dopke, A. Doria, M. T. Dova, A. T. Doyle, E. Drechsler, E. Dreyer, T. Dreyer, Y. Du, J. Duarte-Campderros, F. Dubinin, M. Dubovsky, A. Dubreuil, E. Duchovni, G. Duckeck, A. Ducourthial, O. A. Ducu, D. Duda, A. Dudarev, A. C. Dudder, E. M. Duffield, L. Duflot, M. Dührssen, C. Dülsen, M. Dumancic, A. E. Dumitriu, A. K. Duncan, M. Dunford, A. Duperrin, H. Duran Yildiz, M. Düren, A. Durglishvili, D. Duschinger, B. Dutta, D. Duvnjak, M. Dyndal, S. Dysch, B. S. Dziedzic, C. Eckardt, K. M. Ecker, R. C. Edgar, T. Eifert, G. Eigen, K. Einsweiler, T. Ekelof, M. El Kacimi, R. El Kosseifi, V. Ellajosyula, M. Ellert, F. Ellinghaus, A. A. Elliot, N. Ellis, J. Elmsheuser, M. Elsing, D. Emeliyanov, Y. Enari, J. S. Ennis, M. B. Epland, J. Erdmann, A. Ereditato, S. Errede, M. Escalier, C. Escobar, O. Estrada Pastor, A. I. Etienvre, E. Etzion, H. Evans, A. Ezhilov, M. Ezzi, F. Fabbri, L. Fabbri, V. Fabiani, G. Facini, R. M. Faisca Rodrigues Pereira, R. M. Fakhrutdinov, S. Falciano, P. J. Falke, S. Falke, J. Faltova, Y. Fang, M. Fanti, A. Farbin, A. Farilla, E. M. Farina, T. Farooque, S. Farrell, S. M. Farrington, P. Farthouat, F. Fassi, P. Fassnacht, D. Fassouliotis, M. Faucci Giannelli, A. Favareto, W. J. Fawcett, L. Fayard, O. L. Fedin, W. Fedorko, M. Feickert, S. Feigl, L. Feligioni, C. Feng, E. J. Feng, M. Feng, M. J. Fenton, A. B. Fenyuk, L. Feremenga, J. Ferrando, A. Ferrari, P. Ferrari, R. Ferrari, D. E. Ferreira de Lima, A. Ferrer, D. Ferrere, C. Ferretti, F. Fiedler, A. Filipčič, F. Filthaut, K. D. Finelli, M. C. N. Fiolhais, L. Fiorini, C. Fischer, W. C. Fisher, N. Flaschel, I. Fleck, P. Fleischmann, R. R. M. Fletcher, T. Flick, B. M. Flierl, L. M. Flores, L. R. Flores Castillo, N. Fomin, G. T. Forcolin, A. Formica, F. A. Förster, A. C. Forti, A. G. Foster, D. Fournier, H. Fox, S. Fracchia, P. Francavilla, M. Franchini, S. Franchino, D. Francis, L. Franconi, M. Franklin, M. Frate, M. Fraternali, D. Freeborn, S. M. Fressard-Batraneanu, B. Freund, W. S. Freund, D. Froidevaux, J. A. Frost, C. Fukunaga, E. Fullana Torregrosa, T. Fusayasu, J. Fuster, O. Gabizon, A. Gabrielli, A. Gabrielli, G. P. Gach, S. Gadatsch, P. Gadow, G. Gagliardi, L. G. Gagnon, C. Galea, B. Galhardo, E. J. Gallas, B. J. Gallop, P. Gallus, G. Galster, R. Gamboa Goni, K. K. Gan, S. Ganguly, J. Gao, Y. Gao, Y. S. Gao, C. García, J. E. García Navarro, J. A. García Pascual, M. Garcia-Sciveres, R. W. Gardner, N. Garelli, V. Garonne, K. Gasnikova, A. Gaudiello, G. Gaudio, I. L. Gavrilenko, A. Gavrilyuk, C. Gay, G. Gaycken, E. N. Gazis, C. N. P. Gee, J. Geisen, M. Geisen, M. P. Geisler, K. Gellerstedt, C. Gemme, M. H. Genest, C. Geng, S. Gentile, S. George, D. Gerbaudo, G. Gessner, S. Ghasemi, M. Ghasemi Bostanabad, M. Ghneimat, B. Giacobbe, S. Giagu, N. Giangiacomi, P. Giannetti, A. Giannini, S. M. Gibson, M. Gignac, D. Gillberg, G. Gilles, D. M. Gingrich, M. P. Giordani, F. M. Giorgi, P. F. Giraud, P. Giromini, G. Giugliarelli, D. Giugni, F. Giuli, M. Giulini, S. Gkaitatzis, I. Gkialas, E. L. Gkougkousis, P. Gkountoumis, L. K. Gladilin, C. Glasman, J. Glatzer, P. C. F. Glaysher, A. Glazov, M. Goblirsch-Kolb, J. Godlewski, S. Goldfarb, T. Golling, D. Golubkov, A. Gomes, R. Goncalves Gama, R. Gonçalo, G. Gonella, L. Gonella, A. Gongadze, F. Gonnella, J. L. Gonski, S. González de la Hoz, S. Gonzalez-Sevilla, L. Goossens, P. A. Gorbounov, H. A. Gordon, B. Gorini, E. Gorini, A. Gorišek, A. T. Goshaw, C. Gössling, M. I. Gostkin, C. A. Gottardo, C. R. Goudet, D. Goujdami, A. G. Goussiou, N. Govender, C. Goy, E. Gozani, I. Grabowska-Bold, P. O. J. Gradin, E. C. Graham, J. Gramling, E. Gramstad, S. Grancagnolo, V. Gratchev, P. M. Gravila, F. G. Gravili, C. Gray, H. M. Gray, Z. D. Greenwood, C. Grefe, K. Gregersen, I. M. Gregor, P. Grenier, K. Grevtsov, J. Griffiths, A. A. Grillo, K. Grimm, S. Grinstein, Ph. Gris, J.-F. Grivaz, S. Groh, E. Gross, J. Grosse-Knetter, G. C. Grossi, Z. J. Grout, C. Grud, A. Grummer, L. Guan, W. Guan, J. Guenther, A. Guerguichon, F. Guescini, D. Guest, R. Gugel, B. Gui, T. Guillemin, S. Guindon, U. Gul, C. Gumpert, J. Guo, W. Guo, Y. Guo, Z. Guo, R. Gupta, S. Gurbuz, G. Gustavino, B. J. Gutelman, P. Gutierrez, C. Gutschow, C. Guyot, M. P. Guzik, C. Gwenlan, C. B. Gwilliam, A. Haas, C. Haber, H. K. Hadavand, N. Haddad, A. Hadef, S. Hageböck, M. Hagihara, H. Hakobyan, M. Haleem, J. Haley, G. Halladjian, G. D. Hallewell, K. Hamacher, P. Hamal, K. Hamano, A. Hamilton, G. N. Hamity, K. Han, L. Han, S. Han, K. Hanagaki, M. Hance, D. M. Handl, B. Haney, R. Hankache, P. Hanke, E. Hansen, J. B. Hansen, J. D. Hansen, M. C. Hansen, P. H. Hansen, K. Hara, A. S. Hard, T. Harenberg, S. Harkusha, P. F. Harrison, N. M. Hartmann, Y. Hasegawa, A. Hasib, S. Hassani, S. Haug, R. Hauser, L. Hauswald, L. B. Havener, M. Havranek, C. M. Hawkes, R. J. Hawkings, D. Hayden, C. Hayes, C. P. Hays, J. M. Hays, H. S. Hayward, S. J. Haywood, M. P. Heath, V. Hedberg, L. Heelan, S. Heer, K. K. Heidegger, J. Heilman, S. Heim, T. Heim, B. Heinemann, J. J. Heinrich, L. Heinrich, C. Heinz, J. Hejbal, L. Helary, A. Held, S. Hellesund, S. Hellman, C. Helsens, R. C. W. Henderson, Y. Heng, S. Henkelmann, A. M. Henriques Correia, G. H. Herbert, H. Herde, V. Herget, Y. Hernández Jiménez, H. Herr, M. G. Herrmann, G. Herten, R. Hertenberger, L. Hervas, T. C. Herwig, G. G. Hesketh, N. P. Hessey, J. W. Hetherly, S. Higashino, E. Higón-Rodriguez, K. Hildebrand, E. Hill, J. C. Hill, K. K. Hill, K. H. Hiller, S. J. Hillier, M. Hils, I. Hinchliffe, M. Hirose, D. Hirschbuehl, B. Hiti, O. Hladik, D. R. Hlaluku, X. Hoad, J. Hobbs, N. Hod, M. C. Hodgkinson, A. Hoecker, M. R. Hoeferkamp, F. Hoenig, D. Hohn, D. Hohov, T. R. Holmes, M. Holzbock, M. Homann, S. Honda, T. Honda, T. M. Hong, A. Hönle, B. H. Hooberman, W. H. Hopkins, Y. Horii, P. Horn, A. J. Horton, L. A. Horyn, J-Y. Hostachy, A. Hostiuc, S. Hou, A. Hoummada, J. Howarth, J. Hoya, M. Hrabovsky, J. Hrdinka, I. Hristova, J. Hrivnac, A. Hrynevich, T. Hryn’ova, P. J. Hsu, S.-C. Hsu, Q. Hu, S. Hu, Y. Huang, Z. Hubacek, F. Hubaut, M. Huebner, F. Huegging, T. B. Huffman, E. W. Hughes, M. Huhtinen, R. F. H. Hunter, P. Huo, A. M. Hupe, N. Huseynov, J. Huston, J. Huth, R. Hyneman, G. Iacobucci, G. Iakovidis, I. Ibragimov, L. Iconomidou-Fayard, Z. Idrissi, P. Iengo, R. Ignazzi, O. Igonkina, R. Iguchi, T. Iizawa, Y. Ikegami, M. Ikeno, D. Iliadis, N. Ilic, F. Iltzsche, G. Introzzi, M. Iodice, K. Iordanidou, V. Ippolito, M. F. Isacson, N. Ishijima, M. Ishino, M. Ishitsuka, W. Islam, C. Issever, S. Istin, F. Ito, J. M. Iturbe Ponce, R. Iuppa, A. Ivina, H. Iwasaki, J. M. Izen, V. Izzo, P. Jacka, P. Jackson, R. M. Jacobs, V. Jain, G. Jäkel, K. B. Jakobi, K. Jakobs, S. Jakobsen, T. Jakoubek, D. O. Jamin, D. K. Jana, R. Jansky, J. Janssen, M. Janus, P. A. Janus, G. Jarlskog, N. Javadov, T. Javůrek, M. Javurkova, F. Jeanneau, L. Jeanty, J. Jejelava, A. Jelinskas, P. Jenni, J. Jeong, S. Jézéquel, H. Ji, J. Jia, H. Jiang, Y. Jiang, Z. Jiang, S. Jiggins, F. A. Jimenez Morales, J. Jimenez Pena, S. Jin, A. Jinaru, O. Jinnouchi, H. Jivan, P. Johansson, K. A. Johns, C. A. Johnson, W. J. Johnson, K. Jon-And, R. W. L. Jones, S. D. Jones, S. Jones, T. J. Jones, J. Jongmanns, P. M. Jorge, J. Jovicevic, X. Ju, J. J. Junggeburth, A. Juste Rozas, A. Kaczmarska, M. Kado, H. Kagan, M. Kagan, T. Kaji, E. Kajomovitz, C. W. Kalderon, A. Kaluza, S. Kama, A. Kamenshchikov, L. Kanjir, Y. Kano, V. A. Kantserov, J. Kanzaki, B. Kaplan, L. S. Kaplan, D. Kar, M. J. Kareem, E. Karentzos, S. N. Karpov, Z. M. Karpova, V. Kartvelishvili, A. N. Karyukhin, K. Kasahara, L. Kashif, R. D. Kass, A. Kastanas, Y. Kataoka, C. Kato, J. Katzy, K. Kawade, K. Kawagoe, T. Kawamoto, G. Kawamura, E. F. Kay, V. F. Kazanin, R. Keeler, R. Kehoe, J. S. Keller, E. Kellermann, J. J. Kempster, J. Kendrick, O. Kepka, S. Kersten, B. P. Kerševan, R. A. Keyes, M. Khader, F. Khalil-Zada, A. Khanov, A. G. Kharlamov, T. Kharlamova, A. Khodinov, T. J. Khoo, E. Khramov, J. Khubua, S. Kido, M. Kiehn, C. R. Kilby, S. H. Kim, Y. K. Kim, N. Kimura, O. M. Kind, B. T. King, D. Kirchmeier, J. Kirk, A. E. Kiryunin, T. Kishimoto, D. Kisielewska, V. Kitali, O. Kivernyk, E. Kladiva, T. Klapdor-Kleingrothaus, M. H. Klein, M. Klein, U. Klein, K. Kleinknecht, P. Klimek, A. Klimentov, R. Klingenberg, T. Klingl, T. Klioutchnikova, F. F. Klitzner, P. Kluit, S. Kluth, E. Kneringer, E. B. F. G. Knoops, A. Knue, A. Kobayashi, D. Kobayashi, T. Kobayashi, M. Kobel, M. Kocian, P. Kodys, T. Koffas, E. Koffeman, N. M. Köhler, T. Koi, M. Kolb, I. Koletsou, T. Kondo, N. Kondrashova, K. Köneke, A. C. König, T. Kono, R. Konoplich, V. Konstantinides, N. Konstantinidis, B. Konya, R. Kopeliansky, S. Koperny, K. Korcyl, K. Kordas, A. Korn, I. Korolkov, E. V. Korolkova, O. Kortner, S. Kortner, T. Kosek, V. V. Kostyukhin, A. Kotwal, A. Koulouris, A. Kourkoumeli-Charalampidi, C. Kourkoumelis, E. Kourlitis, V. Kouskoura, A. B. Kowalewska, R. Kowalewski, T. Z. Kowalski, C. Kozakai, W. Kozanecki, A. S. Kozhin, V. A. Kramarenko, G. Kramberger, D. Krasnopevtsev, M. W. Krasny, A. Krasznahorkay, D. Krauss, J. A. Kremer, J. Kretzschmar, P. Krieger, K. Krizka, K. Kroeninger, H. Kroha, J. Kroll, J. Kroll, J. Krstic, U. Kruchonak, H. Krüger, N. Krumnack, M. C. Kruse, T. Kubota, S. Kuday, J. T. Kuechler, S. Kuehn, A. Kugel, F. Kuger, T. Kuhl, V. Kukhtin, R. Kukla, Y. Kulchitsky, S. Kuleshov, Y. P. Kulinich, M. Kuna, T. Kunigo, A. Kupco, T. Kupfer, O. Kuprash, H. Kurashige, L. L. Kurchaninov, Y. A. Kurochkin, M. G. Kurth, E. S. Kuwertz, M. Kuze, J. Kvita, T. Kwan, A. La Rosa, J. L. La Rosa Navarro, L. La Rotonda, F. La Ruffa, C. Lacasta, F. Lacava, J. Lacey, D. P. J. Lack, H. Lacker, D. Lacour, E. Ladygin, R. Lafaye, B. Laforge, T. Lagouri, S. Lai, S. Lammers, W. Lampl, E. Lançon, U. Landgraf, M. P. J. Landon, M. C. Lanfermann, V. S. Lang, J. C. Lange, R. J. Langenberg, A. J. Lankford, F. Lanni, K. Lantzsch, A. Lanza, A. Lapertosa, S. Laplace, J. F. Laporte, T. Lari, F. Lasagni Manghi, M. Lassnig, T. S. Lau, A. Laudrain, M. Lavorgna, A. T. Law, P. Laycock, M. Lazzaroni, B. Le, O. Le Dortz, E. Le Guirriec, E. P. Le Quilleuc, M. LeBlanc, T. LeCompte, F. Ledroit-Guillon, C. A. Lee, G. R. Lee, L. Lee, S. C. Lee, B. Lefebvre, M. Lefebvre, F. Legger, C. Leggett, N. Lehmann, G. Lehmann Miotto, W. A. Leight, A. Leisos, M. A. L. Leite, R. Leitner, D. Lellouch, B. Lemmer, K. J. C. Leney, T. Lenz, B. Lenzi, R. Leone, S. Leone, C. Leonidopoulos, G. Lerner, C. Leroy, R. Les, A. A. J. Lesage, C. G. Lester, M. Levchenko, J. Levêque, D. Levin, L. J. Levinson, D. Lewis, B. Li, C-Q. Li, H. Li, L. Li, Q. Li, Q. Y. Li, S. Li, X. Li, Y. Li, Z. Liang, B. Liberti, A. Liblong, K. Lie, S. Liem, A. Limosani, C. Y. Lin, K. Lin, T. H. Lin, R. A. Linck, B. E. Lindquist, A. L. Lionti, E. Lipeles, A. Lipniacka, M. Lisovyi, T. M. Liss, A. Lister, A. M. Litke, J. D. Little, B. Liu, B. L Liu, H. B. Liu, H. Liu, J. B. Liu, J. K. K. Liu, K. Liu, M. Liu, P. Liu, Y. Liu, Y. L. Liu, Y. W. Liu, M. Livan, A. Lleres, J. Llorente Merino, S. L. Lloyd, C. Y. Lo, F. Lo Sterzo, E. M. Lobodzinska, P. Loch, A. Loesle, K. M. Loew, T. Lohse, K. Lohwasser, M. Lokajicek, B. A. Long, J. D. Long, R. E. Long, L. Longo, K. A. Looper, J. A. Lopez, I. Lopez Paz, A. Lopez Solis, J. Lorenz, N. Lorenzo Martinez, M. Losada, P. J. Lösel, X. Lou, X. Lou, A. Lounis, J. Love, P. A. Love, J. J. Lozano Bahilo, H. Lu, M. Lu, N. Lu, Y. J. Lu, H. J. Lubatti, C. Luci, A. Lucotte, C. Luedtke, F. Luehring, I. Luise, W. Lukas, L. Luminari, B. Lund-Jensen, M. S. Lutz, P. M. Luzi, D. Lynn, R. Lysak, E. Lytken, F. Lyu, V. Lyubushkin, H. Ma, L. L. Ma, Y. Ma, G. Maccarrone, A. Macchiolo, C. M. Macdonald, J. Machado Miguens, D. Madaffari, R. Madar, W. F. Mader, A. Madsen, N. Madysa, J. Maeda, K. Maekawa, S. Maeland, T. Maeno, A. S. Maevskiy, V. Magerl, C. Maidantchik, T. Maier, A. Maio, O. Majersky, S. Majewski, Y. Makida, N. Makovec, B. Malaescu, Pa. Malecki, V. P. Maleev, F. Malek, U. Mallik, D. Malon, C. Malone, S. Maltezos, S. Malyukov, J. Mamuzic, G. Mancini, I. Mandić, J. Maneira, L. Manhaes de Andrade Filho, J. Manjarres Ramos, K. H. Mankinen, A. Mann, A. Manousos, B. Mansoulie, J. D. Mansour, M. Mantoani, S. Manzoni, G. Marceca, L. March, L. Marchese, G. Marchiori, M. Marcisovsky, C. A. Marin Tobon, M. Marjanovic, D. E. Marley, F. Marroquim, Z. Marshall, M. U. F Martensson, S. Marti-Garcia, C. B. Martin, T. A. Martin, V. J. Martin, B. Martin dit Latour, M. Martinez, V. I. Martinez Outschoorn, S. Martin-Haugh, V. S. Martoiu, A. C. Martyniuk, A. Marzin, L. Masetti, T. Mashimo, R. Mashinistov, J. Masik, A. L. Maslennikov, L. H. Mason, L. Massa, P. Massarotti, P. Mastrandrea, A. Mastroberardino, T. Masubuchi, P. Mättig, J. Maurer, B. Maček, S. J. Maxfield, D. A. Maximov, R. Mazini, I. Maznas, S. M. Mazza, N. C. Mc Fadden, G. Mc Goldrick, S. P. Mc Kee, A. McCarn, T. G. McCarthy, L. I. McClymont, E. F. McDonald, J. A. Mcfayden, G. Mchedlidze, M. A. McKay, K. D. McLean, S. J. McMahon, P. C. McNamara, C. J. McNicol, R. A. McPherson, J. E. Mdhluli, Z. A. Meadows, S. Meehan, T. M. Megy, S. Mehlhase, A. Mehta, T. Meideck, B. Meirose, D. Melini, B. R. Mellado Garcia, J. D. Mellenthin, M. Melo, F. Meloni, A. Melzer, S. B. Menary, E. D. Mendes Gouveia, L. Meng, X. T. Meng, A. Mengarelli, S. Menke, E. Meoni, S. Mergelmeyer, C. Merlassino, P. Mermod, L. Merola, C. Meroni, F. S. Merritt, A. Messina, J. Metcalfe, A. S. Mete, C. Meyer, J. Meyer, J-P. Meyer, H. Meyer Zu Theenhausen, F. Miano, R. P. Middleton, L. Mijović, G. Mikenberg, M. Mikestikova, M. Mikuž, M. Milesi, A. Milic, D. A. Millar, D. W. Miller, A. Milov, D. A. Milstead, A. A. Minaenko, M. Miñano Moya, I. A. Minashvili, A. I. Mincer, B. Mindur, M. Mineev, Y. Minegishi, Y. Ming, L. M. Mir, A. Mirto, K. P. Mistry, T. Mitani, J. Mitrevski, V. A. Mitsou, A. Miucci, P. S. Miyagawa, A. Mizukami, J. U. Mjörnmark, T. Mkrtchyan, M. Mlynarikova, T. Moa, K. Mochizuki, P. Mogg, S. Mohapatra, S. Molander, R. Moles-Valls, M. C. Mondragon, K. Mönig, J. Monk, E. Monnier, A. Montalbano, J. Montejo Berlingen, F. Monticelli, S. Monzani, R. W. Moore, N. Morange, D. Moreno, M. Moreno Llácer, P. Morettini, M. Morgenstern, S. Morgenstern, D. Mori, T. Mori, M. Morii, M. Morinaga, V. Morisbak, A. K. Morley, G. Mornacchi, A. P. Morris, J. D. Morris, L. Morvaj, P. Moschovakos, M. Mosidze, H. J. Moss, J. Moss, K. Motohashi, R. Mount, E. Mountricha, E. J. W. Moyse, S. Muanza, F. Mueller, J. Mueller, R. S. P. Mueller, D. Muenstermann, P. Mullen, G. A. Mullier, F. J. Munoz Sanchez, P. Murin, W. J. Murray, A. Murrone, M. Muškinja, C. Mwewa, A. G. Myagkov, J. Myers, M. Myska, B. P. Nachman, O. Nackenhorst, K. Nagai, K. Nagano, Y. Nagasaka, K. Nagata, M. Nagel, E. Nagy, A. M. Nairz, Y. Nakahama, K. Nakamura, T. Nakamura, I. Nakano, H. Nanjo, F. Napolitano, R. F. Naranjo Garcia, R. Narayan, D. I. Narrias Villar, I. Naryshkin, T. Naumann, G. Navarro, R. Nayyar, H. A. Neal, P. Y. Nechaeva, T. J. Neep, A. Negri, M. Negrini, S. Nektarijevic, C. Nellist, M. E. Nelson, S. Nemecek, P. Nemethy, M. Nessi, M. S. Neubauer, M. Neumann, P. R. Newman, T. Y. Ng, Y. S. Ng, H. D. N. Nguyen, T. Nguyen Manh, E. Nibigira, R. B. Nickerson, R. Nicolaidou, J. Nielsen, N. Nikiforou, V. Nikolaenko, I. Nikolic-Audit, K. Nikolopoulos, P. Nilsson, Y. Ninomiya, A. Nisati, N. Nishu, R. Nisius, I. Nitsche, T. Nitta, T. Nobe, Y. Noguchi, M. Nomachi, I. Nomidis, M. A. Nomura, T. Nooney, M. Nordberg, N. Norjoharuddeen, T. Novak, O. Novgorodova, R. Novotny, L. Nozka, K. Ntekas, E. Nurse, F. Nuti, F. G. Oakham, H. Oberlack, T. Obermann, J. Ocariz, A. Ochi, I. Ochoa, J. P. Ochoa-Ricoux, K. O’Connor, S. Oda, S. Odaka, S. Oerdek, A. Oh, S. H. Oh, C. C. Ohm, H. Oide, H. Okawa, Y. Okazaki, Y. Okumura, T. Okuyama, A. Olariu, L. F. Oleiro Seabra, S. A. Olivares Pino, D. Oliveira Damazio, J. L. Oliver, M. J. R. Olsson, A. Olszewski, J. Olszowska, D. C. O’Neil, A. Onofre, K. Onogi, P. U. E. Onyisi, H. Oppen, M. J. Oreglia, Y. Oren, D. Orestano, E. C. Orgill, N. Orlando, A. A. O’Rourke, R. S. Orr, B. Osculati, V. O’Shea, R. Ospanov, G. Otero y Garzon, H. Otono, M. Ouchrif, F. Ould-Saada, A. Ouraou, Q. Ouyang, M. Owen, R. E. Owen, V. E. Ozcan, N. Ozturk, J. Pacalt, H. A. Pacey, K. Pachal, A. Pacheco Pages, L. Pacheco Rodriguez, C. Padilla Aranda, S. Pagan Griso, M. Paganini, G. Palacino, S. Palazzo, S. Palestini, M. Palka, D. Pallin, I. Panagoulias, C. E. Pandini, J. G. Panduro Vazquez, P. Pani, G. Panizzo, L. Paolozzi, T. D. Papadopoulou, K. Papageorgiou, A. Paramonov, D. Paredes Hernandez, S. R. Paredes Saenz, B. Parida, A. J. Parker, K. A. Parker, M. A. Parker, F. Parodi, J. A. Parsons, U. Parzefall, V. R. Pascuzzi, J. M. P. Pasner, E. Pasqualucci, S. Passaggio, F. Pastore, P. Pasuwan, S. Pataraia, J. R. Pater, A. Pathak, T. Pauly, B. Pearson, M. Pedersen, L. Pedraza Diaz, R. Pedro, S. V. Peleganchuk, O. Penc, C. Peng, H. Peng, B. S. Peralva, M. M. Perego, A. P. Pereira Peixoto, D. V. Perepelitsa, F. Peri, L. Perini, H. Pernegger, S. Perrella, V. D. Peshekhonov, K. Peters, R. F. Y. Peters, B. A. Petersen, T. C. Petersen, E. Petit, A. Petridis, C. Petridou, P. Petroff, E. Petrolo, M. Petrov, F. Petrucci, M. Pettee, N. E. Pettersson, A. Peyaud, R. Pezoa, T. Pham, F. H. Phillips, P. W. Phillips, G. Piacquadio, E. Pianori, A. Picazio, M. A. Pickering, R. Piegaia, J. E. Pilcher, A. D. Pilkington, M. Pinamonti, J. L. Pinfold, M. Pitt, M-A. Pleier, V. Pleskot, E. Plotnikova, D. Pluth, P. Podberezko, R. Poettgen, R. Poggi, L. Poggioli, I. Pogrebnyak, D. Pohl, I. Pokharel, G. Polesello, A. Poley, A. Policicchio, R. Polifka, A. Polini, C. S. Pollard, V. Polychronakos, D. Ponomarenko, L. Pontecorvo, G. A. Popeneciu, D. M. Portillo Quintero, S. Pospisil, K. Potamianos, I. N. Potrap, C. J. Potter, H. Potti, T. Poulsen, J. Poveda, T. D. Powell, M. E. Pozo Astigarraga, P. Pralavorio, S. Prell, D. Price, M. Primavera, S. Prince, N. Proklova, K. Prokofiev, F. Prokoshin, S. Protopopescu, J. Proudfoot, M. Przybycien, A. Puri, P. Puzo, J. Qian, Y. Qin, A. Quadt, M. Queitsch-Maitland, A. Qureshi, P. Rados, F. Ragusa, G. Rahal, J. A. Raine, S. Rajagopalan, A. Ramirez Morales, T. Rashid, S. Raspopov, M. G. Ratti, D. M. Rauch, F. Rauscher, S. Rave, B. Ravina, I. Ravinovich, J. H. Rawling, M. Raymond, A. L. Read, N. P. Readioff, M. Reale, D. M. Rebuzzi, A. Redelbach, G. Redlinger, R. Reece, R. G. Reed, K. Reeves, L. Rehnisch, J. Reichert, A. Reiss, C. Rembser, H. Ren, M. Rescigno, S. Resconi, E. D. Resseguie, S. Rettie, E. Reynolds, O. L. Rezanova, P. Reznicek, R. Richter, S. Richter, E. Richter-Was, O. Ricken, M. Ridel, P. Rieck, C. J. Riegel, O. Rifki, M. Rijssenbeek, A. Rimoldi, M. Rimoldi, L. Rinaldi, G. Ripellino, B. Ristić, E. Ritsch, I. Riu, J. C. Rivera Vergara, F. Rizatdinova, E. Rizvi, C. Rizzi, R. T. Roberts, S. H. Robertson, A. Robichaud-Veronneau, D. Robinson, J. E. M. Robinson, A. Robson, E. Rocco, C. Roda, Y. Rodina, S. Rodriguez Bosca, A. Rodriguez Perez, D. Rodriguez Rodriguez, A. M. Rodríguez Vera, S. Roe, C. S. Rogan, O. Røhne, R. Röhrig, C. P. A. Roland, J. Roloff, A. Romaniouk, M. Romano, N. Rompotis, M. Ronzani, L. Roos, S. Rosati, K. Rosbach, P. Rose, N-A. Rosien, E. Rossi, E. Rossi, L. P. Rossi, L. Rossini, J. H. N. Rosten, R. Rosten, M. Rotaru, J. Rothberg, D. Rousseau, D. Roy, A. Rozanov, Y. Rozen, X. Ruan, F. Rubbo, F. Rühr, A. Ruiz-Martinez, Z. Rurikova, N. A. Rusakovich, H. L. Russell, J. P. Rutherfoord, E. M. Rüttinger, Y. F. Ryabov, M. Rybar, G. Rybkin, S. Ryu, A. Ryzhov, G. F. Rzehorz, P. Sabatini, G. Sabato, S. Sacerdoti, H. F-W. Sadrozinski, R. Sadykov, F. Safai Tehrani, P. Saha, M. Sahinsoy, A. Sahu, M. Saimpert, M. Saito, T. Saito, H. Sakamoto, A. Sakharov, D. Salamani, G. Salamanna, J. E. Salazar Loyola, D. Salek, P. H. Sales De Bruin, D. Salihagic, A. Salnikov, J. Salt, D. Salvatore, F. Salvatore, A. Salvucci, A. Salzburger, J. Samarati, D. Sammel, D. Sampsonidis, D. Sampsonidou, J. Sánchez, A. Sanchez Pineda, H. Sandaker, C. O. Sander, M. Sandhoff, C. Sandoval, D. P. C. Sankey, M. Sannino, Y. Sano, A. Sansoni, C. Santoni, H. Santos, I. Santoyo Castillo, A. Sapronov, J. G. Saraiva, O. Sasaki, K. Sato, E. Sauvan, P. Savard, N. Savic, R. Sawada, C. Sawyer, L. Sawyer, C. Sbarra, A. Sbrizzi, T. Scanlon, J. Schaarschmidt, P. Schacht, B. M. Schachtner, D. Schaefer, L. Schaefer, J. Schaeffer, S. Schaepe, U. Schäfer, A. C. Schaffer, D. Schaile, R. D. Schamberger, N. Scharmberg, V. A. Schegelsky, D. Scheirich, F. Schenck, M. Schernau, C. Schiavi, S. Schier, L. K. Schildgen, Z. M. Schillaci, E. J. Schioppa, M. Schioppa, K. E. Schleicher, S. Schlenker, K. R. Schmidt-Sommerfeld, K. Schmieden, C. Schmitt, S. Schmitt, S. Schmitz, U. Schnoor, L. Schoeffel, A. Schoening, E. Schopf, M. Schott, J. F. P. Schouwenberg, J. Schovancova, S. Schramm, A. Schulte, H-C. Schultz-Coulon, M. Schumacher, B. A. Schumm, Ph. Schune, A. Schwartzman, T. A. Schwarz, H. Schweiger, Ph. Schwemling, R. Schwienhorst, A. Sciandra, G. Sciolla, M. Scornajenghi, F. Scuri, F. Scutti, L. M. Scyboz, J. Searcy, C. D. Sebastiani, P. Seema, S. C. Seidel, A. Seiden, T. Seiss, J. M. Seixas, G. Sekhniaidze, K. Sekhon, S. J. Sekula, N. Semprini-Cesari, S. Sen, S. Senkin, C. Serfon, L. Serin, L. Serkin, M. Sessa, H. Severini, F. Sforza, A. Sfyrla, E. Shabalina, J. D. Shahinian, N. W. Shaikh, L. Y. Shan, R. Shang, J. T. Shank, M. Shapiro, A. S. Sharma, A. Sharma, P. B. Shatalov, K. Shaw, S. M. Shaw, A. Shcherbakova, Y. Shen, N. Sherafati, A. D. Sherman, P. Sherwood, L. Shi, S. Shimizu, C. O. Shimmin, M. Shimojima, I. P. J. Shipsey, S. Shirabe, M. Shiyakova, J. Shlomi, A. Shmeleva, D. Shoaleh Saadi, M. J. Shochet, S. Shojaii, D. R. Shope, S. Shrestha, E. Shulga, P. Sicho, A. M. Sickles, P. E. Sidebo, E. Sideras Haddad, O. Sidiropoulou, A. Sidoti, F. Siegert, Dj. Sijacki, J. Silva, M. Silva, M. V. Silva Oliveira, S. B. Silverstein, L. Simic, S. Simion, E. Simioni, M. Simon, R. Simoniello, P. Sinervo, N. B. Sinev, M. Sioli, G. Siragusa, I. Siral, S. Yu. Sivoklokov, J. Sjölin, M. B. Skinner, P. Skubic, M. Slater, T. Slavicek, M. Slawinska, K. Sliwa, R. Slovak, V. Smakhtin, B. H. Smart, J. Smiesko, N. Smirnov, S. Yu. Smirnov, Y. Smirnov, L. N. Smirnova, O. Smirnova, J. W. Smith, M. N. K. Smith, R. W. Smith, M. Smizanska, K. Smolek, A. A. Snesarev, I. M. Snyder, S. Snyder, R. Sobie, A. M. Soffa, A. Soffer, A. Søgaard, D. A. Soh, G. Sokhrannyi, C. A. Solans Sanchez, M. Solar, E. Yu. Soldatov, U. Soldevila, A. A. Solodkov, A. Soloshenko, O. V. Solovyanov, V. Solovyev, P. Sommer, H. Son, W. Song, A. Sopczak, F. Sopkova, D. Sosa, C. L. Sotiropoulou, S. Sottocornola, R. Soualah, A. M. Soukharev, D. South, B. C. Sowden, S. Spagnolo, M. Spalla, M. Spangenberg, F. Spanò, D. Sperlich, F. Spettel, T. M. Spieker, R. Spighi, G. Spigo, L. A. Spiller, D. P. Spiteri, M. Spousta, A. Stabile, R. Stamen, S. Stamm, E. Stanecka, R. W. Stanek, C. Stanescu, B. Stanislaus, M. M. Stanitzki, B. Stapf, S. Stapnes, E. A. Starchenko, G. H. Stark, J. Stark, S. H Stark, P. Staroba, P. Starovoitov, S. Stärz, R. Staszewski, M. Stegler, P. Steinberg, B. Stelzer, H. J. Stelzer, O. Stelzer-Chilton, H. Stenzel, T. J. Stevenson, G. A. Stewart, M. C. Stockton, G. Stoicea, P. Stolte, S. Stonjek, A. Straessner, J. Strandberg, S. Strandberg, M. Strauss, P. Strizenec, R. Ströhmer, D. M. Strom, R. Stroynowski, A. Strubig, S. A. Stucci, B. Stugu, J. Stupak, N. A. Styles, D. Su, J. Su, S. Suchek, Y. Sugaya, M. Suk, V. V. Sulin, D. M. S. Sultan, S. Sultansoy, T. Sumida, S. Sun, X. Sun, K. Suruliz, C. J. E. Suster, M. R. Sutton, S. Suzuki, M. Svatos, M. Swiatlowski, S. P. Swift, A. Sydorenko, I. Sykora, T. Sykora, D. Ta, K. Tackmann, J. Taenzer, A. Taffard, R. Tafirout, E. Tahirovic, N. Taiblum, H. Takai, R. Takashima, E. H. Takasugi, K. Takeda, T. Takeshita, Y. Takubo, M. Talby, A. A. Talyshev, J. Tanaka, M. Tanaka, R. Tanaka, R. Tanioka, B. B. Tannenwald, S. Tapia Araya, S. Tapprogge, A. Tarek Abouelfadl Mohamed, S. Tarem, G. Tarna, G. F. Tartarelli, P. Tas, M. Tasevsky, T. Tashiro, E. Tassi, A. Tavares Delgado, Y. Tayalati, A. C. Taylor, A. J. Taylor, G. N. Taylor, P. T. E. Taylor, W. Taylor, A. S. Tee, P. Teixeira-Dias, H. Ten Kate, P. K. Teng, J. J. Teoh, F. Tepel, S. Terada, K. Terashi, J. Terron, S. Terzo, M. Testa, R. J. Teuscher, S. J. Thais, T. Theveneaux-Pelzer, F. Thiele, J. P. Thomas, A. S. Thompson, P. D. Thompson, L. A. Thomsen, E. Thomson, Y. Tian, R. E. Ticse Torres, V. O. Tikhomirov, Yu. A. Tikhonov, S. Timoshenko, P. Tipton, S. Tisserant, K. Todome, S. Todorova-Nova, S. Todt, J. Tojo, S. Tokár, K. Tokushuku, E. Tolley, K. G. Tomiwa, M. Tomoto, L. Tompkins, K. Toms, B. Tong, P. Tornambe, E. Torrence, H. Torres, E. Torró Pastor, C. Tosciri, J. Toth, F. Touchard, D. R. Tovey, C. J. Treado, T. Trefzger, F. Tresoldi, A. Tricoli, I. M. Trigger, S. Trincaz-Duvoid, M. F. Tripiana, W. Trischuk, B. Trocmé, A. Trofymov, C. Troncon, M. Trovatelli, F. Trovato, L. Truong, M. Trzebinski, A. Trzupek, F. Tsai, J. C-L. Tseng, P. V. Tsiareshka, N. Tsirintanis, V. Tsiskaridze, E. G. Tskhadadze, I. I. Tsukerman, V. Tsulaia, S. Tsuno, D. Tsybychev, Y. Tu, A. Tudorache, V. Tudorache, T. T. Tulbure, A. N. Tuna, S. Turchikhin, D. Turgeman, I. Turk Cakir, R. Turra, P. M. Tuts, E. Tzovara, G. Ucchielli, I. Ueda, M. Ughetto, F. Ukegawa, G. Unal, A. Undrus, G. Unel, F. C. Ungaro, Y. Unno, K. Uno, J. Urban, P. Urquijo, P. Urrejola, G. Usai, J. Usui, L. Vacavant, V. Vacek, B. Vachon, K. O. H. Vadla, A. Vaidya, C. Valderanis, E. Valdes Santurio, M. Valente, S. Valentinetti, A. Valero, L. Valéry, R. A. Vallance, A. Vallier, J. A. Valls Ferrer, T. R. Van Daalen, W. Van Den Wollenberg, H. Van der Graaf, P. Van Gemmeren, J. Van Nieuwkoop, I. Van Vulpen, M. Vanadia, W. Vandelli, A. Vaniachine, P. Vankov, R. Vari, E. W. Varnes, C. Varni, T. Varol, D. Varouchas, K. E. Varvell, G. A. Vasquez, J. G. Vasquez, F. Vazeille, D. Vazquez Furelos, T. Vazquez Schroeder, J. Veatch, V. Vecchio, L. M. Veloce, F. Veloso, S. Veneziano, A. Ventura, M. Venturi, N. Venturi, V. Vercesi, M. Verducci, C. M. Vergel Infante, W. Verkerke, A. T. Vermeulen, J. C. Vermeulen, M. C. Vetterli, N. Viaux Maira, M. Vicente Barreto Pinto, I. Vichou, T. Vickey, O. E. Vickey Boeriu, G. H. A. Viehhauser, S. Viel, L. Vigani, M. Villa, M. Villaplana Perez, E. Vilucchi, M. G. Vincter, V. B. Vinogradov, A. Vishwakarma, C. Vittori, I. Vivarelli, S. Vlachos, M. Vogel, P. Vokac, G. Volpi, S. E. von Buddenbrock, E. Von Toerne, V. Vorobel, K. Vorobev, M. Vos, J. H. Vossebeld, N. Vranjes, M. Vranjes Milosavljevic, V. Vrba, M. Vreeswijk, T. Šfiligoj, R. Vuillermet, I. Vukotic, T. Ženiš, L. Živković, P. Wagner, W. Wagner, J. Wagner-Kuhr, H. Wahlberg, S. Wahrmund, K. Wakamiya, V. M. Walbrecht, J. Walder, R. Walker, S. D. Walker, W. Walkowiak, V. Wallangen, A. M. Wang, C. Wang, F. Wang, H. Wang, H. Wang, J. Wang, J. Wang, P. Wang, Q. Wang, R.-J. Wang, R. Wang, R. Wang, S. M. Wang, W. T. Wang, W. Wang, W. X. Wang, Y. Wang, Z. Wang, C. Wanotayaroj, A. Warburton, C. P. Ward, D. R. Wardrope, A. Washbrook, P. M. Watkins, A. T. Watson, M. F. Watson, G. Watts, S. Watts, B. M. Waugh, A. F. Webb, S. Webb, C. Weber, M. S. Weber, S. A. Weber, S. M. Weber, A. R. Weidberg, B. Weinert, J. Weingarten, M. Weirich, C. Weiser, P. S. Wells, T. Wenaus, T. Wengler, S. Wenig, N. Wermes, M. D. Werner, P. Werner, M. Wessels, T. D. Weston, K. Whalen, N. L. Whallon, A. M. Wharton, A. S. White, A. White, M. J. White, R. White, D. Whiteson, B. W. Whitmore, F. J. Wickens, W. Wiedenmann, M. Wielers, C. Wiglesworth, L. A. M. Wiik-Fuchs, A. Wildauer, F. Wilk, H. G. Wilkens, L. J. Wilkins, H. H. Williams, S. Williams, C. Willis, S. Willocq, J. A. Wilson, I. Wingerter-Seez, E. Winkels, F. Winklmeier, O. J. Winston, B. T. Winter, M. Wittgen, M. Wobisch, A. Wolf, T. M. H. Wolf, R. Wolff, M. W. Wolter, H. Wolters, V. W. S. Wong, N. L. Woods, S. D. Worm, B. K. Wosiek, K. W. Woźniak, K. Wraight, M. Wu, S. L. Wu, X. Wu, Y. Wu, T. R. Wyatt, B. M. Wynne, S. Xella, Z. Xi, L. Xia, D. Xu, H. Xu, L. Xu, T. Xu, W. Xu, B. Yabsley, S. Yacoob, K. Yajima, D. P. Yallup, D. Yamaguchi, Y. Yamaguchi, A. Yamamoto, T. Yamanaka, F. Yamane, M. Yamatani, T. Yamazaki, Y. Yamazaki, Z. Yan, H. J. Yang, H. T. Yang, S. Yang, Y. Yang, Z. Yang, W-M. Yao, Y. C. Yap, Y. Yasu, E. Yatsenko, J. Ye, S. Ye, I. Yeletskikh, E. Yigitbasi, E. Yildirim, K. Yorita, K. Yoshihara, C. J. S. Young, C. Young, J. Yu, J. Yu, X. Yue, S. P. Y. Yuen, B. Zabinski, G. Zacharis, E. Zaffaroni, R. Zaidan, A. M. Zaitsev, N. Zakharchuk, J. Zalieckas, S. Zambito, D. Zanzi, D. R. Zaripovas, S. V. Zeißner, C. Zeitnitz, G. Zemaityte, J. C. Zeng, Q. Zeng, O. Zenin, D. Zerwas, M. Zgubič, D. F. Zhang, D. Zhang, F. Zhang, G. Zhang, H. Zhang, J. Zhang, L. Zhang, L. Zhang, M. Zhang, P. Zhang, R. Zhang, R. Zhang, X. Zhang, Y. Zhang, Z. Zhang, P. Zhao, X. Zhao, Y. Zhao, Z. Zhao, A. Zhemchugov, B. Zhou, C. Zhou, L. Zhou, M. S. Zhou, M. Zhou, N. Zhou, Y. Zhou, C. G. Zhu, H. L. Zhu, H. Zhu, J. Zhu, Y. Zhu, X. Zhuang, K. Zhukov, V. Zhulanov, A. Zibell, D. Zieminska, N. I. Zimine, S. Zimmermann, Z. Zinonos, M. Zinser, M. Ziolkowski, G. Zobernig, A. Zoccoli, K. Zoch, T. G. Zorbas, R. Zou, M. Zur Nedden, L. Zwalinski, ATLAS Collaboration

**Affiliations:** 1 Department of Physics, University of Adelaide, Adelaide, Australia; 2 Physics Department, SUNY Albany, Albany, NY USA; 3 Department of Physics, University of Alberta, Edmonton, AB Canada; 4 Department of Physics, Ankara University, Ankara, Turkey; 5 Istanbul Aydin University, Istanbul, Turkey; 6 Division of Physics, TOBB University of Economics and Technology, Ankara, Turkey; 7 LAPP, Université Grenoble Alpes, Université Savoie Mont Blanc, CNRS/IN2P3, Annecy, France; 8 High Energy Physics Division, Argonne National Laboratory, Argonne, IL USA; 9 Department of Physics, University of Arizona, Tucson, AZ USA; 10 Department of Physics, University of Texas at Arlington, Arlington, TX USA; 11 Physics Department, National and Kapodistrian University of Athens, Athens, Greece; 12 Physics Department, National Technical University of Athens, Zografou, Greece; 13 Department of Physics, University of Texas at Austin, Austin, TX USA; 14 Faculty of Engineering and Natural Sciences, Bahcesehir University, Istanbul, Turkey; 15 Faculty of Engineering and Natural Sciences, Istanbul Bilgi University, Istanbul, Turkey; 16 Department of Physics, Bogazici University, Istanbul, Turkey; 17 Department of Physics Engineering, Gaziantep University, Gaziantep, Turkey; 18 Institute of Physics, Azerbaijan Academy of Sciences, Baku, Azerbaijan; 19 Institut de Física d’Altes Energies (IFAE), Barcelona Institute of Science and Technology, Barcelona, Spain; 20 Institute of High Energy Physics, Chinese Academy of Sciences, Beijing, China; 21 Physics Department, Tsinghua University, Beijing, China; 22 Department of Physics, Nanjing University, Nanjing, China; 23 University of Chinese Academy of Science (UCAS), Beijing, China; 24 Institute of Physics, University of Belgrade, Belgrade, Serbia; 25 Department for Physics and Technology, University of Bergen, Bergen, Norway; 26 Physics Division, Lawrence Berkeley National Laboratory and University of California, Berkeley, CA USA; 27 Institut für Physik, Humboldt Universität zu Berlin, Berlin, Germany; 28 Albert Einstein Center for Fundamental Physics and Laboratory for High Energy Physics, University of Bern, Bern, Switzerland; 29 School of Physics and Astronomy, University of Birmingham, Birmingham, UK; 30 Centro de Investigaciónes, Universidad Antonio Nariño, Bogotá, Colombia; 31 Dipartimento di Fisica e Astronomia, Università di Bologna, Bologna, Italy; 32 INFN Sezione di Bologna, Bologna, Italy; 33 Physikalisches Institut, Universität Bonn, Bonn, Germany; 34 Department of Physics, Boston University, Boston, MA USA; 35 Department of Physics, Brandeis University, Waltham, MA USA; 36 Transilvania University of Brasov, Brasov, Romania; 37 Horia Hulubei National Institute of Physics and Nuclear Engineering, Bucharest, Romania; 38 Department of Physics, Alexandru Ioan Cuza University of Iasi, Iasi, Romania; 39 Physics Department, National Institute for Research and Development of Isotopic and Molecular Technologies, Cluj-Napoca, Romania; 40 University Politehnica Bucharest, Bucharest, Romania; 41 West University in Timisoara, Timisoara, Romania; 42 Faculty of Mathematics, Physics and Informatics, Comenius University, Bratislava, Slovakia; 43 Department of Subnuclear Physics, Institute of Experimental Physics of the Slovak Academy of Sciences, Kosice, Slovak Republic; 44 Physics Department, Brookhaven National Laboratory, Upton, NY USA; 45 Departamento de Física, Universidad de Buenos Aires, Buenos Aires, Argentina; 46 Cavendish Laboratory, University of Cambridge, Cambridge, UK; 47 Department of Physics, University of Cape Town, Cape Town, South Africa; 48 Department of Mechanical Engineering Science, University of Johannesburg, Johannesburg, South Africa; 49 School of Physics, University of the Witwatersrand, Johannesburg, South Africa; 50 Department of Physics, Carleton University, Ottawa, ON Canada; 51 Faculté des Sciences Ain Chock, Réseau Universitaire de Physique des Hautes Energies-Université Hassan II, Casablanca, Morocco; 52 Centre National de l’Energie des Sciences Techniques Nucleaires (CNESTEN), Rabat, Morocco; 53 Faculté des Sciences Semlalia, Université Cadi Ayyad, LPHEA-Marrakech, Marrakech, Morocco; 54 Faculté des Sciences, Université Mohamed Premier and LPTPM, Oujda, Morocco; 55 Faculté des sciences, Université Mohammed V, Rabat, Morocco; 56 CERN, Geneva, Switzerland; 57 Enrico Fermi Institute, University of Chicago, Chicago, IL USA; 58 LPC, Université Clermont Auvergne, CNRS/IN2P3, Clermont-Ferrand, France; 59 Nevis Laboratory, Columbia University, Irvington, NY USA; 60 Niels Bohr Institute, University of Copenhagen, Copenhagen, Denmark; 61 Dipartimento di Fisica, Università della Calabria, Rende, Italy; 62 INFN Gruppo Collegato di Cosenza, Laboratori Nazionali di Frascati, Frascati, Italy; 63 Physics Department, Southern Methodist University, Dallas, TX USA; 64 Physics Department, University of Texas at Dallas, Richardson, TX USA; 65 Department of Physics, Stockholm University, Stockholm, Sweden; 66 Oskar Klein Centre, Stockholm, Sweden; 67 Deutsches Elektronen-Synchrotron DESY, Hamburg and Zeuthen, Germany; 68 Lehrstuhl für Experimentelle Physik IV, Technische Universität Dortmund, Dortmund, Germany; 69 Institut für Kern- und Teilchenphysik, Technische Universität Dresden, Dresden, Germany; 70 Department of Physics, Duke University, Durham, NC USA; 71 SUPA-School of Physics and Astronomy, University of Edinburgh, Edinburgh, UK; 72 INFN e Laboratori Nazionali di Frascati, Frascati, Italy; 73 Physikalisches Institut, Albert-Ludwigs-Universität Freiburg, Freiburg, Germany; 74 II. Physikalisches Institut, Georg-August-Universität Göttingen, Göttingen, Germany; 75 Département de Physique Nucléaire et Corpusculaire, Université de Genève, Geneva, Switzerland; 76 Dipartimento di Fisica, Università di Genova, Genoa, Italy; 77 INFN Sezione di Genova, Genoa, Italy; 78 II. Physikalisches Institut, Justus-Liebig-Universität Giessen, Giessen, Germany; 79 SUPA-School of Physics and Astronomy, University of Glasgow, Glasgow, UK; 80 LPSC, Université Grenoble Alpes, CNRS/IN2P3, Grenoble INP, Grenoble, France; 81 Laboratory for Particle Physics and Cosmology, Harvard University, Cambridge, MA USA; 82 Department of Modern Physics and State Key Laboratory of Particle Detection and Electronics, University of Science and Technology of China, Hefei, China; 83 Institute of Frontier and Interdisciplinary Science and Key Laboratory of Particle Physics and Particle Irradiation (MOE), Shandong University, Qingdao, China; 84 School of Physics and Astronomy, Shanghai Jiao Tong University, KLPPAC-MoE, SKLPPC, Shanghai, China; 85 Tsung-Dao Lee Institute, Shanghai, China; 86 Kirchhoff-Institut für Physik, Ruprecht-Karls-Universität Heidelberg, Heidelberg, Germany; 87 Physikalisches Institut, Ruprecht-Karls-Universität Heidelberg, Heidelberg, Germany; 88 Faculty of Applied Information Science, Hiroshima Institute of Technology, Hiroshima, Japan; 89 Department of Physics, Chinese University of Hong Kong, Shatin, NT Hong Kong; 90 Department of Physics, University of Hong Kong, Hong Kong, China; 91 Department of Physics and Institute for Advanced Study, Hong Kong University of Science and Technology, Clear Water Bay, Kowloon, Hong Kong, China; 92 Department of Physics, National Tsing Hua University, Hsinchu, Taiwan; 93 Department of Physics, Indiana University, Bloomington, IN USA; 94 INFN Gruppo Collegato di Udine, Sezione di Trieste, Udine, Italy; 95 ICTP, Trieste, Italy; 96 Dipartimento di Chimica, Fisica e Ambiente, Università di Udine, Udine, Italy; 97 INFN Sezione di Lecce, Lecce, Italy; 98 Dipartimento di Matematica e Fisica, Università del Salento, Lecce, Italy; 99 INFN Sezione di Milano, Milan, Italy; 100 Dipartimento di Fisica, Università di Milano, Milan, Italy; 101 INFN Sezione di Napoli, Naples, Italy; 102 Dipartimento di Fisica, Università di Napoli, Naples, Italy; 103 INFN Sezione di Pavia, Pavia, Italy; 104 Dipartimento di Fisica, Università di Pavia, Pavia, Italy; 105 INFN Sezione di Pisa, Pisa, Italy; 106 Dipartimento di Fisica E. Fermi, Università di Pisa, Pisa, Italy; 107 INFN Sezione di Roma, Rome, Italy; 108 Dipartimento di Fisica, Sapienza Università di Roma, Rome, Italy; 109 INFN Sezione di Roma Tor Vergata, Rome, Italy; 110 Dipartimento di Fisica, Università di Roma Tor Vergata, Rome, Italy; 111 INFN Sezione di Roma Tre, Rome, Italy; 112 Dipartimento di Matematica e Fisica, Università Roma Tre, Rome, Italy; 113 INFN-TIFPA, Povo, Italy; 114 Università degli Studi di Trento, Trento, Italy; 115 Institut für Astro- und Teilchenphysik, Leopold-Franzens-Universität, Innsbruck, Austria; 116 University of Iowa, Iowa City, IA USA; 117 Department of Physics and Astronomy, Iowa State University, Ames, IA USA; 118 Joint Institute for Nuclear Research, Dubna, Russia; 119 Departamento de Engenharia Elétrica, Universidade Federal de Juiz de Fora (UFJF), Juiz de Fora, Brazil; 120 Universidade Federal do Rio De Janeiro COPPE/EE/IF, Rio de Janeiro, Brazil; 121 Universidade Federal de São João del Rei (UFSJ), São João del Rei, Brazil; 122 Instituto de Física, Universidade de São Paulo, São Paulo, Brazil; 123 KEK, High Energy Accelerator Research Organization, Tsukuba, Japan; 124 Graduate School of Science, Kobe University, Kobe, Japan; 125 Faculty of Physics and Applied Computer Science, AGH University of Science and Technology, Kraków, Poland; 126 Marian Smoluchowski Institute of Physics, Jagiellonian University, Kraków, Poland; 127 Institute of Nuclear Physics Polish Academy of Sciences, Kraków, Poland; 128 Faculty of Science, Kyoto University, Kyoto, Japan; 129 Kyoto University of Education, Kyoto, Japan; 130 Research Center for Advanced Particle Physics and Department of Physics, Kyushu University, Fukuoka, Japan; 131 Instituto de Física La Plata, Universidad Nacional de La Plata and CONICET, La Plata, Argentina; 132 Physics Department, Lancaster University, Lancaster, UK; 133 Oliver Lodge Laboratory, University of Liverpool, Liverpool, UK; 134 Department of Experimental Particle Physics, Jožef Stefan Institute and Department of Physics, University of Ljubljana, Ljubljana, Slovenia; 135 School of Physics and Astronomy, Queen Mary University of London, London, UK; 136 Department of Physics, Royal Holloway University of London, Egham, UK; 137 Department of Physics and Astronomy, University College London, London, UK; 138 Louisiana Tech University, Ruston, LA USA; 139 Fysiska institutionen, Lunds universitet, Lund, Sweden; 140 Centre de Calcul de l’Institut National de Physique Nucléaire et de Physique des Particules (IN2P3), Villeurbanne, France; 141 Departamento de Física Teorica C-15 and CIAFF, Universidad Autónoma de Madrid, Madrid, Spain; 142 Institut für Physik, Universität Mainz, Mainz, Germany; 143 School of Physics and Astronomy, University of Manchester, Manchester, UK; 144 CPPM, Aix-Marseille Université, CNRS/IN2P3, Marseille, France; 145 Department of Physics, University of Massachusetts, Amherst, MA USA; 146 Department of Physics, McGill University, Montreal, QC Canada; 147 School of Physics, University of Melbourne, Parkville, VIC Australia; 148 Department of Physics, University of Michigan, Ann Arbor, MI USA; 149 Department of Physics and Astronomy, Michigan State University, East Lansing, MI USA; 150 B.I. Stepanov Institute of Physics, National Academy of Sciences of Belarus, Minsk, Belarus; 151 Research Institute for Nuclear Problems of Byelorussian State University, Minsk, Belarus; 152 Group of Particle Physics, University of Montreal, Montreal, QC Canada; 153 P.N. Lebedev Physical Institute of the Russian Academy of Sciences, Moscow, Russia; 154 Institute for Theoretical and Experimental Physics (ITEP), Moscow, Russia; 155 National Research Nuclear University MEPhI, Moscow, Russia; 156 D.V. Skobeltsyn Institute of Nuclear Physics, M.V. Lomonosov Moscow State University, Moscow, Russia; 157 Fakultät für Physik, Ludwig-Maximilians-Universität München, Munich, Germany; 158 Max-Planck-Institut für Physik (Werner-Heisenberg-Institut), Munich, Germany; 159 Nagasaki Institute of Applied Science, Nagasaki, Japan; 160 Graduate School of Science and Kobayashi-Maskawa Institute, Nagoya University, Nagoya, Japan; 161 Department of Physics and Astronomy, University of New Mexico, Albuquerque, NM USA; 162 Institute for Mathematics, Astrophysics and Particle Physics, Radboud University Nijmegen/Nikhef, Nijmegen, The Netherlands; 163 Nikhef National Institute for Subatomic Physics, University of Amsterdam, Amsterdam, The Netherlands; 164 Department of Physics, Northern Illinois University, De Kalb, IL USA; 165 Budker Institute of Nuclear Physics, SB RAS, Novosibirsk, Russia; 166 Novosibirsk State University, Novosibirsk, Russia; 167 Department of Physics, New York University, New York, NY USA; 168 Ohio State University, Columbus, OH USA; 169 Faculty of Science, Okayama University, Okayama, Japan; 170 Homer L. Dodge Department of Physics and Astronomy, University of Oklahoma, Norman, OK USA; 171 Department of Physics, Oklahoma State University, Stillwater, OK USA; 172 Palacký University, RCPTM, Joint Laboratory of Optics, Olomouc, Czech Republic; 173 Center for High Energy Physics, University of Oregon, Eugene, OR USA; 174 LAL, Université Paris-Sud, CNRS/IN2P3, Université Paris-Saclay, Orsay, France; 175 Graduate School of Science, Osaka University, Osaka, Japan; 176 Department of Physics, University of Oslo, Oslo, Norway; 177 Department of Physics, Oxford University, Oxford, UK; 178 LPNHE, Sorbonne Université, Paris Diderot Sorbonne Paris Cité, CNRS/IN2P3, Paris, France; 179 Department of Physics, University of Pennsylvania, Philadelphia, PA USA; 180 Konstantinov Nuclear Physics Institute of National Research Centre “Kurchatov Institute”, PNPI, St. Petersburg, Russia; 181 Department of Physics and Astronomy, University of Pittsburgh, Pittsburgh, PA USA; 182 Laboratório de Instrumentação e Física Experimental de Partículas-LIP, Lisbon, Portugal; 183 Departamento de Física, Faculdade de Ciências, Universidade de Lisboa, Lisbon, Portugal; 184 Departamento de Física, Universidade de Coimbra, Coimbra, Portugal; 185 Centro de Física Nuclear da Universidade de Lisboa, Lisbon, Portugal; 186 Departamento de Física, Universidade do Minho, Braga, Portugal; 187 Departamento de Física Teorica y del Cosmos, Universidad de Granada, Granada, Spain; 188 Dep Física and CEFITEC of Faculdade de Ciências e Tecnologia, Universidade Nova de Lisboa, Caparica, Portugal; 189 Institute of Physics, Academy of Sciences of the Czech Republic, Prague, Czech Republic; 190 Czech Technical University in Prague, Prague, Czech Republic; 191 Faculty of Mathematics and Physics, Charles University, Prague, Czech Republic; 192 State Research Center Institute for High Energy Physics, NRC KI, Protvino, Russia; 193 Particle Physics Department, Rutherford Appleton Laboratory, Didcot, UK; 194 IRFU, CEA, Université Paris-Saclay, Gif-sur-Yvette, France; 195 Santa Cruz Institute for Particle Physics, University of California Santa Cruz, Santa Cruz, CA USA; 196 Departamento de Física, Pontificia Universidad Católica de Chile, Santiago, Chile; 197 Departamento de Física, Universidad Técnica Federico Santa María, Valparaíso, Chile; 198 Department of Physics, University of Washington, Seattle, WA USA; 199 Department of Physics and Astronomy, University of Sheffield, Sheffield, UK; 200 Department of Physics, Shinshu University, Nagano, Japan; 201 Department Physik, Universität Siegen, Siegen, Germany; 202 Department of Physics, Simon Fraser University, Burnaby, BC Canada; 203 SLAC National Accelerator Laboratory, Stanford, CA USA; 204 Physics Department, Royal Institute of Technology, Stockholm, Sweden; 205 Departments of Physics and Astronomy, Stony Brook University, Stony Brook, NY USA; 206 Department of Physics and Astronomy, University of Sussex, Brighton, UK; 207 School of Physics, University of Sydney, Sydney, Australia; 208 Institute of Physics, Academia Sinica, Taipei, Taiwan; 209 E. Andronikashvili Institute of Physics, Iv. Javakhishvili Tbilisi State University, Tbilisi, Georgia; 210 High Energy Physics Institute, Tbilisi State University, Tbilisi, Georgia; 211 Department of Physics, Technion, Israel Institute of Technology, Haifa, Israel; 212 Raymond and Beverly Sackler School of Physics and Astronomy, Tel Aviv University, Tel Aviv, Israel; 213 Department of Physics, Aristotle University of Thessaloniki, Thessaloniki, Greece; 214 International Center for Elementary Particle Physics and Department of Physics, University of Tokyo, Tokyo, Japan; 215 Graduate School of Science and Technology, Tokyo Metropolitan University, Tokyo, Japan; 216 Department of Physics, Tokyo Institute of Technology, Tokyo, Japan; 217 Tomsk State University, Tomsk, Russia; 218 Department of Physics, University of Toronto, Toronto, ON Canada; 219 TRIUMF, Vancouver, BC Canada; 220 Department of Physics and Astronomy, York University, Toronto, ON Canada; 221 Division of Physics and Tomonaga Center for the History of the Universe, Faculty of Pure and Applied Sciences, University of Tsukuba, Tsukuba, Japan; 222 Department of Physics and Astronomy, Tufts University, Medford, MA USA; 223 Department of Physics and Astronomy, University of California Irvine, Irvine, CA USA; 224 Department of Physics and Astronomy, University of Uppsala, Uppsala, Sweden; 225 Department of Physics, University of Illinois, Urbana, IL USA; 226 Instituto de Física Corpuscular (IFIC), Centro Mixto Universidad de Valencia - CSIC, Valencia, Spain; 227 Department of Physics, University of British Columbia, Vancouver, BC Canada; 228 Department of Physics and Astronomy, University of Victoria, Victoria, BC Canada; 229 Fakultät für Physik und Astronomie, Julius-Maximilians-Universität Würzburg, Würzburg, Germany; 230 Department of Physics, University of Warwick, Coventry, UK; 231 Waseda University, Tokyo, Japan; 232 Department of Particle Physics, Weizmann Institute of Science, Rehovot, Israel; 233 Department of Physics, University of Wisconsin, Madison, WI USA; 234 Fakultät für Mathematik und Naturwissenschaften, Fachgruppe Physik, Bergische Universität Wuppertal, Wuppertal, Germany; 235 Department of Physics, Yale University, New Haven, CT USA; 236 Yerevan Physics Institute, Yerevan, Armenia; 237 CERN, 1211 Geneva 23, Switzerland

## Abstract

A search for doubly charged scalar bosons decaying into *W* boson pairs is presented. It uses a data sample from proton–proton collisions corresponding to an integrated luminosity of 36.1 fb$^\mathrm {-1}$ collected by the ATLAS detector at the LHC at a centre-of-mass energy of 13 TeV in 2015 and 2016. This search is guided by a model that includes an extension of the Higgs sector through a scalar triplet, leading to a rich phenomenology that includes doubly charged scalar bosons $H^{\pm \pm }$. Those bosons are produced in pairs in proton–proton collisions and decay predominantly into electroweak gauge bosons $H^{\pm \pm }\rightarrow W^{\pm } W^{\pm }$. Experimental signatures with several leptons, missing transverse energy and jets are explored. No significant deviations from the Standard Model predictions are found. The parameter space of the benchmark model is excluded at 95% confidence level for $H^{\pm \pm }$ bosons with masses between 200 and 220 GeV.

## Introduction

An extension of the scalar sector of the Standard Model (SM) is possible in the context of type II seesaw models [1], originally conceived to explain the smallness of the neutrino masses. In the model investigated in this paper, the scalar sector includes a hypercharge $$Y=2$$ scalar triplet, $$\Delta $$, in addition to the SM scalar doublet *H* [2, 3]. Electroweak symmetry breaking (EWSB) is achieved if the neutral components of *H* and $$\Delta $$ acquire vacuum expectation values, $$v_{d}$$ and $$v_{t}$$ respectively. After the EWSB, the mixing between these fields results in seven scalar bosons: $$H^{\pm \pm }$$, $$H^{\pm }$$, $$A^{0}$$ (CP odd), $$H^{0}$$ (CP even), $$h^{0}$$ (CP even). A small mixing between the CP-even scalars allows $$h^{0}$$ to have the expected properties of the SM Higgs boson. In addition, the triplet-neutrino Yukawa term provides non-zero neutrino masses proportional to the vacuum expectation value of the triplet $$v_{t}$$. Constraints from electroweak precision measurements lead to an upper bound on $$v_t $$ of around 1 GeV. This range is significantly lower than the electroweak scale and matches the need for small values suggested by the natural association of $$v_t $$ with the neutrino masses.

The assumption of a non-zero $$v_t $$, of the order of a hundred MeV, opens the possibility for the doubly charged boson to decay into a pair of same-sign *W* bosons, $$H^{\pm \pm }\rightarrow W^\pm W^\pm $$, while the leptonic decays $$H^{\pm \pm }\rightarrow \ell ^\pm \ell ^\pm $$ are suppressed with increasing $$v_t $$ [4, 5]. Extensive searches for leptonic decays $$H^{\pm \pm }\rightarrow \ell ^\pm \ell ^\pm $$ have been performed at various colliders [6–11], where $$H^{\pm \pm }$$ bosons with masses up to about 800 GeV have been excluded. Moreover, searches for $$H^{\pm \pm }\rightarrow W^\pm W^\pm $$ decays have been performed by the CMS Collaboration in the context of single $$H^{\pm \pm }$$ production through vector-boson fusion at large $$v_t $$ (of order of tens of GeV) [12, 13] for a model with two Higgs triplets [14]. For that model, a custodial symmetry avoids large contributions to the electroweak precision observables [15]. In contrast, the $$H^{\pm \pm }\rightarrow W^\pm W^\pm $$ decay mode has not been directly searched for so far for small values of $$v_t $$, where the vector-boson fusion is suppressed.

The present paper focuses on the phenomenology of doubly charged scalar bosons $H^{\pm \pm }$ that can be produced in pairs at the Large Hadron Collider (LHC) and decay into *W* bosons. The triplet vacuum expectation value is taken to be $v_t =0.1$ GeVsuch that only the $H^{\pm \pm }\rightarrow W^\pm W^\pm$ decays are relevant, leading to final states with four *W* bosons. The mixing between the CP-even scalars is taken to be $10^{-4}$ and the remaining five Yukawa parameters in the potential are adjusted to obtain a given $H^{\pm \pm }$ mass hypothesis while requiring $h^{0}$ to have a mass of 125 GeV. The corresponding cross-section calculation is performed for on-shell *W* bosons, and therefore only the region $m_{H^{\pm \pm }} > 200$ GeV is considered in the present analysis.

The four-boson final states are identified by the presence of light charged leptons (electrons or muons), missing transverse momentum, and jets. The analysis uses three final states defined according to the number of light leptons: same-sign (SS) dilepton channel ($2\ell ^\text {ss}$), trilepton channel ($3\ell$) and four-lepton channel ($4\ell$). Similar final states were used for other searches for new phenomena in ATLAS [16–18]. However, the previously searched signal topologies differ significantly from those targeted in the present analysis and a dedicated event selection optimisation is therefore applied.

This paper includes a description of the experimental set-up in Sect. 2, followed by a description of the simulation used in the analysis in Sect. 3. The event selection and background estimations for the three explored signatures are described in Sect. 4. The signal region optimisation is described in Sect.  5. The systematic uncertainties are presented in Sect. 6. The results are shown in Sect. 7, followed by the conclusions in Sect. 8.

## ATLAS detector

The ATLAS experiment [19] at the LHC is a multipurpose particle detector with a forward–backward symmetric cylindrical geometry and a near $4\pi$ coverage in solid angle.^1^ It consists of an inner tracking detector surrounded by a superconducting solenoid providing a 2 T axial magnetic field, electromagnetic and hadronic calorimeters, and a muon spectrometer. The inner tracking detector, covering the pseudorapidity range $|\eta | < 2.5$, consists of silicon pixel and silicon microstrip tracking detectors inside a transition-radiation tracker that covers $|\eta | < 2.0$. It includes, for the $\sqrt{s} = 13$ TeVrunning period, a newly installed innermost pixel layer, the insertable *B*-layer [20]. Lead/liquid-argon (LAr) sampling calorimeters provide electromagnetic (EM) energy measurements for $|\eta | < 2.5$ with high granularity and longitudinal segmentation. A hadronic calorimeter consisting of steel and scintillator tiles covers the central pseudorapidity range ($|\eta | < 1.7$). The endcap and forward regions are instrumented with LAr calorimeters for EM and hadronic energy measurements up to $|\eta | = 4.9$. The muon spectrometer surrounds the calorimeters and is based on three large air-core toroid superconducting magnets with eight coils each. It includes a system of precision tracking chambers ($|\eta | < 2.7$) and fast detectors for triggering ($|\eta | < 2.4$). A two-level trigger system is used to select events [21]. The first-level trigger is implemented in hardware and uses a subset of the detector information to reduce the accepted rate to a design maximum of 100 kHz. This is followed by a software-based trigger with a sustained average accepted event rate of about 1 kHz.

## Data and simulation

The data sample collected by the ATLAS Collaboration at $\sqrt{s}=13$ TeV during 2015 and 2016 was used. After the application of beam and data quality requirements, the integrated luminosity is $36.1\, \hbox {fb}^{-1}$.

Monte Carlo (MC) simulation samples were produced for signal and background processes using the full ATLAS detector simulation [22] based on Geant4 [23] or, for selected smaller backgrounds and some of the signal samples, a fast simulation using a parameterisation of the calorimeter response and Geant4 for the tracking system [24]. To simulate the effects of additional *pp* collisions in the same and nearby bunch crossings (pile-up), additional interactions were generated using Pythia  8.186 [25, 26] with a set of tuned parameters for the underlying event, referred to as the A2 tune [27], and the MSTW2008LO set of parton distribution functions (PDF) [28], and overlaid on the simulated hard-scatter event. The simulated events were reweighted to match the distribution of the number of interactions per bunch crossing observed in the data and were reconstructed using the same procedure as for the data.

The signal events containing $H^{\pm \pm }$ pairs were simulated with the CalcHEP generator version 3.4 [29], which is at leading order in QCD, using the Lagrangian described in Ref. [3] and the PDF set CTEQ6L1 [30, 31]. The modelling of the parton showering and hadronisation of these events was performed using PYTHIA 8.186 [25, 26] with the A14 tune [32]. Event samples for the process $pp \rightarrow H^{\pm \pm }H^{\mp \mp }\rightarrow W^\pm W^\pm W^\mp W^\mp$ were simulated for $m_{H^{\pm \pm }}$ in the range from 200 to 700 GeV with steps of 100 GeV. The production cross-section decreases rapidly with $m_{H^{\pm \pm }}$ and is 80.7 fb for $m_{H^{\pm \pm }}=200~\text {GeV}$, 5.0 fb for $m_{H^{\pm \pm }}=400~\text {GeV}$, and 0.35 fb for $m_{H^{\pm \pm }}=700~\text {GeV}$. Next-to-leading order (NLO) corrections [33] in QCD were applied, which increase these cross-sections by a factor 1.25. The fast detector simulation was used for the samples corresponding to $m_{H^{\pm \pm }} > 500$ GeV.

**Table 1:** Configurations used for event generation of background processes

Process	Event generator	ME order	Parton shower	PDF	Tune
*VV*, *qqVV*, *VVV*	Sherpa 2.1.1 [35]	MEPS NLO	Sherpa 2.1.1	CT10 [36]	Sherpa 2.1.1 default
$t\bar{t}H$	MG5_aMC [37]	NLO	Pythia 8 [26]	NNPDF 3.0 NLO [38]	A14 [32]
*VH*	Pythia 8	LO	Pythia 8	NNPDF 2.3 LO	A14
*tHqb*	MG5_aMC	LO	Pythia 8	CT10	A14
*tHW*	MG5_aMC	NLO	Herwig++ [39]	CT10	UE-EE-5 [40]
$t\bar{t} W$, $t\bar{t} (Z/\gamma ^*)$	MG5_aMC	NLO	Pythia 8	NNPDF 3.0 NLO	A14
$t (Z/\gamma ^*)$	MG5_aMC	LO	Pythia 6 [25]	CTEQ6L1 [30, 31]	Perugia2012 [41]
$t W (Z/\gamma ^*)$	MG5_aMC	NLO	Pythia 8	NNPDF 2.3 LO	A14
$t\bar{t} t, t\bar{t} t\bar{t}$	MG5_aMC	LO	Pythia 8	NNPDF 2.3 LO	A14
$t\bar{t} W^+ W^-$	MG5_aMC	LO	Pythia 8	NNPDF 2.3 LO	A14
$V\gamma$	Sherpa 2.2	MEPS NLO	Sherpa 2.2	NNPDF 3.0 NLO	Sherpa 2.2 default
*s*-, *t*-channel, *Wt* single top	Powheg-Box v 2 [42, 43]	NLO	Pythia 6	CT10/CTEQ6L1	Perugia2012

If only one PDF is shown, the same is used for both the matrix element (ME) and parton shower generators; if two are shown, the first is used for the matrix element calculation and the second for the parton shower. *V* refers to the production of an electroweak boson (*W* or $Z/\gamma ^*$). “Tune” refers to the underlying-event tune of the parton shower generator. “MG5_aMC” refers to MadGraph5_aMC@NLO 2.2.1; “Pythia 6” refers to version 6.427; “Pythia 8” refers to version 8.1; “Herwig++ ” refers to version 2.7. The samples have heavy flavour hadron decays modelled by EvtGen1.2.0 [34], except for samples generated with Sherpa

The SM background processes were simulated using the MC event generator programs and configurations shown in Table 1. The production of *VV*, *VVqq*, and *VVV* (where *V* denotes a vector boson *W* or *Z* and *qq* labels the vector-boson fusion production mechanism) was simulated with a NLO QCD matrix element computed by Sherpa and matched to the Sherpa parton shower. The main background contribution in the $2\ell ^\text {ss}$ and $3\ell$ channels is from *WZ* production, for which the total cross-section prediction is $48.2\pm 1.1$ pb [44]. The main contribution to the $4\ell$ topology is from *ZZ* production with a total cross-section of $16.9\pm 0.6$ pb [45, 46], which is suppressed by requiring significant missing transverse momentum in these events. The MC samples used to simulate $t\bar{t}H$, $t\bar{t}V$, *VV* and $t\bar{t}$ are described in more detail in Refs. [47–49].

The simulated SM contributions in each of the channels considered are separated into prompt-lepton and fake-lepton contributions, depending on the source of the reconstructed leptons at generator level. The processes that contain only reconstructed charged leptons originating from prompt leptonic decays of *W* and *Z* bosons are classified as a prompt-lepton contribution, while processes with at least one of the reconstructed leptons being a misidentified hadron or photon, or a lepton from hadron decays constitute the fake-lepton contribution. The simulated events are not used to evaluate the background originating from charge-misidentified leptons for the $2\ell ^\text {ss}$ channel and fake leptons for the $2\ell ^\text {ss}$ and $3\ell$ channels. These are estimated in general using data-driven methods because they are not well modelled by simulations. This is the case in particular for $Z \rightarrow \ell ^+\ell ^-$, $W \rightarrow \ell \nu$ and $t\bar{t}$ processes. The background process $V\gamma$ can contribute if electrons originating from the photon conversion are selected. This contribution is found to be small and adequately modelled, so it is estimated using the MC simulation. For the $4\ell$ channel, the background from fake leptons is small and the data-driven methods are not applicable due to the low number of events available, so the MC simulation is used to estimate both the prompt-lepton and the fake-lepton contributions.

## Event selection and background estimates

### Event reconstruction

Interaction vertices originating from *pp* collisions are reconstructed using at least two tracks with transverse momentum $p_{\mathrm {T}} > 0.4$ GeV, and required to be consistent with the beam-spot envelope. The primary vertex is identified as the vertex with the largest sum of squares of the transverse momenta from associated tracks [50].

Electrons are reconstructed as tracks in the inner detector matched to clusters in the electromagnetic calorimeter, within the region of pseudorapidity $|\eta | < 2.47$ [51]. The candidates in the transition region between the barrel and the endcap calorimeters ($1.37< |\eta | < 1.52$) are removed. Only those electron candidates with transverse momentum greater than 10 GeVare considered. The electron identification is based on a multivariate likelihood-based discriminant that uses the shower shapes in the electromagnetic calorimeter and the associated track properties measured in the inner detector. In particular, the *loose* and *tight* identification working points, described in Ref. [51], are used, providing electron identification efficiencies of approximately 95% and 78–$90\%$ (depending on $p_{\mathrm {T}}$ and $\eta$), respectively. In order to reduce contributions from converted photons and hadron decays, the longitudinal impact parameter of the electron track relative to the selected event primary vertex, multiplied by the sine of the polar angle, $|z_{0}\sin \theta |$, is required to be less than 0.5 mm. The transverse impact parameter divided by its uncertainty, $|d_{0}|/\sigma (d_{0})$, is required to be less than five. The identification algorithm is complemented by an isolation requirement, based on the energy in a cone around the electron candidate calculated using either charged tracks or calorimetric deposits. The calorimeter- and track-based isolation criteria are applied jointly to suppress fake electrons.

Muon candidates are reconstructed by combining tracks formed in the inner detector and in the muon spectrometer, within the region of pseudorapidity $|\eta | <$ 2.5 [52]. Only those muon candidates with transverse momentum greater than 10 GeVare considered. A muon candidate is required to satisfy *loose* or *tight* identification criteria which are defined in Ref. [52], and which have efficiencies of approximately 98% and 92%, respectively. Similarly to electrons, isolation criteria complement the identification requirements. The impact parameters must satisfy $|z_{0}\sin \theta | < 0.5$ mm and $|d_{0}|/\sigma (d_{0}) < 3$ when selecting muons.

Combining the selection criteria mentioned above, two types of lepton requirements are used for both the electrons and muons: type *T* (for *tight*) and *L* (for *loose*). The type *T* leptons are a subset of the type *L*.

Jets are reconstructed from topological clusters [53] of energy deposits in the calorimeters using the anti-$k_t$ algorithm [54, 55] with a radius parameter of $R = 0.4$. Only jets with $p_{\mathrm {T}} > 25$ GeVand $|\eta | < 2.5$ are considered. In order to suppress jets arising from pile-up collisions, jets with $p_{\mathrm {T}} < 60$ GeVand $|\eta | <2.4$ must have a sizeable fraction of their tracks matched to the selected primary vertex [56]. Jets containing *b*-hadrons are identified (*b*-tagged) via a multi-variate discriminant combining information from the impact parameters of displaced tracks with topological properties of secondary and tertiary decay vertices reconstructed within the jet [57]. The *b*-tagging algorithm used for this search has an average efficiency of 70% to identify *b*-jets with $p_{\mathrm {T}} > 20$ GeVand $|\eta | < 2.5$ in simulated $t\bar{t}$ events.

To avoid object double counting, an overlap removal procedure is applied to resolve ambiguities among electrons, muons, and jets in the final state. Any electron candidate sharing an inner detector track with a muon candidate is removed. Jets within $\Delta R = 0.2$ of an electron, as well as jets with less than three tracks within $\Delta R = 0.2$ of a muon candidate are discarded. Any remaining electron candidate within $\Delta R = 0.4$ of a jet is discarded. Any remaining muon candidate within $\Delta R = 0.04 + 10/p_{\mathrm {T}} ^{\mu } (\text {GeV})$ of a jet is discarded.

The missing transverse momentum, with magnitude $E_\text {T}^\text {miss}$, is defined as the negative vector sum of the transverse momenta of all identified leptons and jets and the remaining unclustered energy of the event, which is estimated from tracks associated with the primary vertex but not assigned to any physics object [58].

### Event preselection

Candidate events are selected using triggers that require at least one electron or one muon to pass various thresholds of $p_{\mathrm {T}}$ [21]. The higher thresholds are applied with looser lepton identification and/or isolation requirements in order to ensure efficiencies close to 100% for leptons with transverse momentum above 30 GeV.

The signal topologies studied in this search involve the presence of at least two leptons of the same charge and are classified as explained above in three mutually exclusive categories: $2\ell ^\text {ss}$, $3\ell$ and $4\ell$ channels. The $2\ell ^\text {ss}$ channel targets signal events where the two same-sign *W* bosons from one of the doubly charged Higgs boson decays leptonically, while the two *W* bosons from the other doubly charged Higgs boson decay hadronically. In the $3\ell$ channel, one *W* boson decays hadronically and in the $4\ell$ channel, all *W* bosons decay leptonically. All channels present significant $E_{\text {T}}^{\text {miss}}$ corresponding to the neutrinos from leptonic *W* boson decays. In the $2\ell ^\text {ss}$ and $4\ell$ channels, jets from *W* boson decays originate from the first- and second-generation quarks, and therefore lead to events without *b*-jets. The event selection is divided into two steps: the preselection and the signal region selection.

The preselection requirements are summarised in Table 2. The electrons (muons) are selected in the pseudorapidity range $|\eta | < 2.47\ (2.5)$ with a transverse momentum of at least 10 GeV, satisfying the type *L* requirement. Events are selected only if the absolute value of the sum of charges of the leptons is two, one and zero for the ${2\ell ^{ss}}$, $3\ell$ and $4\ell$ channels, respectively. At least one of the leptons is required to have $p_{\mathrm {T}}$ $> 30$ GeV to ensure a high trigger efficiency. To reduce the fake-lepton contamination in the $2\ell ^\text {ss}$ channel, the second highest $p_{\mathrm {T}}$ (subleading) lepton is required to have $p_{\mathrm {T}}$ $> 20$ GeVand both leptons are required to be of type *T*. Similarly in the $3\ell$ channel, each lepton in the pair of leptons of the same sign, which is expected to suffer more from fake-lepton contamination, is required to have $p_{\mathrm {T}}$ $> 20$ GeV and to both be of type *T*. In the $2\ell ^\text {ss}$ and $4\ell$ channels, the leptons are labelled by descending $p_{\mathrm {T}}$, and are denoted by $\ell _{1,2,...}$. The ranking follows a different logic for the $3\ell$ channel: the lepton that has a charge opposite to the total lepton charge is denoted as $\ell _0$, while the same-sign leptons are denoted by $\ell _1$ and $\ell _2$, ranked by increasing distance to $\ell _0$ in the $\eta$–$\phi$ plane.

Further preselection requirements are based on $E_{\text {T}}^{\text {miss}}$, the jet multiplicity $N_\text {jets}$ and the number of jets tagged as *b*-jets $N_{b\text {-jet}}$. Moreover, in order to reduce the background from *Z* bosons and neutral mesons decaying into same-flavour opposite-sign leptons (SFOS), the invariant mass of such lepton pairs is required to be greater than 12 (15) GeV for the $3\ell$ ($4\ell$) channel and to have an invariant mass that is not compatible with the *Z* boson. For the $2\ell ^\text {ss}$ channel, the *Z* boson invariant mass veto is also applied to $e^\pm e^\pm$ events, in order to reduce the contributions originating from electron charge misidentification.

After this preselection, 562 data events are selected in the $2\ell ^\text {ss}$ channel, 392 events in the $3\ell$ channel, and 44 events in the $4\ell$ channel.

**Table 2:** The preselection criteria for the three analysis channels

Selection criteria	$2\ell ^\text {ss}$	$3\ell$	$4\ell$
Trigger	At least one lepton with $p_{\mathrm {T}} ^{\ell }>30$ GeV that fulfils the requirements of single-lepton triggers		
$N_\ell$(*L*-type, $p_{\mathrm {T}} >10$ GeV, $|\eta _{\ell }|<2.47$)	2	3	4
$N_\ell$(*T*-type, $p_{\mathrm {T}} >10$ GeV, $|\eta _{\ell }|<2.47$)	2	2 ($\ell _{1,2}$)	−
$|\sum Q_\ell |$	2	1	0
Lepton $p_{\mathrm {T}}$ threshold	$p_{\mathrm {T}} ^{\ell _1,\ell _2}>30, 20$ GeV	$p_{\mathrm {T}} ^{\ell _0,\ell _1,\ell _2}>10, 20, 20$ GeV	$p_{\mathrm {T}} ^{\ell _1,\ell _2,\ell _3,\ell _4}>10$ GeV
$E_{\text {T}}^{\text {miss}}$	$>70$ GeV	$>30$ GeV	$>30$ GeV
$N_\text {jets}$	$\ge 3$	$\ge 2$	–
*b*-jet veto	$N_{b \text {-jet}}=0$	$N_{b \text {-jet}}=0$	$N_{b \text {-jet}}=0$
Low SFOS $m_{\ell \ell }$ veto	–	$m_{\ell ^\pm \ell ^\mp }>15~\text {GeV}$	$m_{\ell ^\pm \ell ^\mp }>12~\text {GeV}$
*Z* boson decays veto	$|m_{e^\pm e^\pm }-m_Z|>10~\text {GeV}$	$|m_{\ell ^\pm \ell ^\mp }-m_Z|>10~\text {GeV}$	

The leptons are ordered by decreasing $p_{\mathrm {T}}$ ($\ell _1,\ell _2,\ldots$) in the $2\ell ^\text {ss}$ and $4\ell$ channels, while for the $3\ell$ channel $\ell _1,\ell _2$ denote the same-sign leptons and $\ell _0$ the lepton with a charge opposite to the total lepton charge. $Q_\ell$ denotes the charge of each lepton

### Background estimate

The background processes containing only prompt selected leptons are estimated with MC simulations normalised to the most precise cross-section calculation (see Sect. 3). Further contributions originate from non-prompt and mismeasured leptons. The procedures used to estimate those contributions are described in the following.

#### Charge misidentification

In the $2\ell ^\text {ss}$ channel, a background contribution is expected from events with opposite-sign lepton pairs when the charge of one of the leptons is misidentified, while the background contribution from charge misidentification is negligible for $3\ell$ and $4\ell$ channels. In the transverse momentum domain relevant for this analysis, charge misidentification is only significant for electrons and is due mainly to bremsstrahlung interactions with the inner detector material. The radiated photon produces an $e^+e^-$ pair near the original electron trajectory leading to a charge identification confusion.

The misidentification rate is measured using a large data sample of dilepton events originating mainly from $Z\rightarrow e^+e^-$ decays selected by two type *T* electrons with an invariant mass between 80 and 100 GeV. The sample contains mostly opposite-sign dileptons, with a small fraction of same-sign dileptons. The fraction of same-sign dilepton events is used to extract the charge-misidentification rate as a function of electron $p_{\mathrm {T}}$ and $\eta$. This rate is found to range between 0.02% and 10%, where large values are obtained at large rapidities where the amount of material is higher. The statistical error of this estimate is taken as systematic uncertainty of the charge misidentification rate. The background from fake leptons in both the opposite-sign and same-sign samples is estimated using sidebands around the *Z* boson mass peak. Its impact on the charge misidentification rate is about 2% and is included in the systematic uncertainty.

The background from charge misidentification in a given region is estimated using a data control sample selected with the same criteria as the nominal sample but with opposite-sign dilepton pairs, where at least one lepton is an electron, weighted by the probability that the charge of the electron(s) is misidentified.

#### Fake-lepton contributions

The composition of the fake-lepton background varies considerably among the analysis channels. Therefore, the methods to estimate the fake-lepton contributions are different for the $2\ell ^\text {ss}$, $3\ell$ and $4\ell$ channels. The contribution from fake leptons for the $2\ell ^\text {ss}$ and $3\ell$ channels are estimated using the fake-factor method, while the simulation prediction corrected with data-driven scale factors is used for the $4\ell$ channels. Those methods involve various fake-enriched control samples that are summarised in Table 3 and described below.

**Table 3:** The selection criteria defining the fake-enriched control regions used to determine the fake factors for the $2\ell ^\text {ss}$ and $3\ell$ channel and the MC scale factors for the $4\ell$ channel

Sample	$2\ell ^\text {ss}$	$3\ell$	$4\ell$-Z	$4\ell$-T
$N_{\ell }$ (type *L*)	2	3	3	3
$|\sum Q_\ell |$	2	1	1	1
$p_{\mathrm {T}} ^{\ell }$	$>30,20$ GeV	$>10,20,20$ GeV	$>10,10,10$ GeV	$>10,10,10$ GeV
$N_\text {jets}$	$\ge 3$	1	$1 \; \mathrm {or} \;2$	$1 \; \mathrm {or} \; 2$
$N_{b \text {-jet}}$	0	−	−	−
$p_{\mathrm {T}} ^{jet}$	$>25$ GeV	$>25$ GeV	$>25$ GeV	$>30 (25)$ GeV
*Z*-window	$|m_{ee}^\text {ss}- m_{Z}| > 10$ GeV	$|m_{\ell \ell }^\text {os}- m_{Z}| > 10$ GeV	$|m_{\ell \ell }^\text {os}- m_{Z}| < 10$ GeV	No same-flavour
				opposite-sign lepton pair
$m_{\ell \ell }^\text {os}$	−	$>15$ GeV	−	−
$E_{\text {T}}^{\text {miss}}$	$<70$ GeV	−	$<50$ GeV	−
$m_\text {T}$	−	−	$<50$ GeV	−

The symbol “−” means no requirement. The transverse mass $m_\text {T}$, used for the $4\ell$-Z region to reduce the *WZ* contributions, is calculated as the invariant mass of the vector sum of transverse momentum of the fake-lepton candidate and the missing transverse momentum

**Fake-lepton contribution estimate for the** $2\ell ^\text {ss}$ **channel** The fake-factor method assumes that the fake-lepton contribution in a nominal region, which can be the preselection or the signal region, can be computed using an extrapolation factor that is referred to as a fake factor, and is denoted as $\theta$ in the following. The fake factor is multiplied by the number of events containing fake leptons in a region with the same selection criteria as the nominal region, except that at least one of the leptons is required to satisfy the type *L* but not the type *T* identification criteria. That lepton is denoted by ,  or collectively  in the following.

The fake factors are calculated in fake-enriched control regions with kinematic selections designed to enhance their content in fake leptons. In the case of the $2\ell ^\text {ss}$ channel ($2\ell ^\text {ss}$ column in Table 3), this is achieved by requiring low $E_{\text {T}}^{\text {miss}}$. The fake factor is defined as the number of fake-lepton events in the fake-enriched region where all selected leptons pass the type *T* identification, divided by the number of fake-lepton events in the same region but where one of the selected leptons is of type .

The muon fake factor is thus computed in the fake-enriched region, where a pair of same-sign muons was selected, as follows:1where $N^\mathrm {Data, C}_\mathrm {\mu \mu }$ and  are the number of events where both muons are of type *T*, and where one is of type *T* and the other of type , respectively. The prompt-lepton contributions $N^\mathrm {Prompt}$, which are estimated using MC simulation, are subtracted from data event yields to obtain a pure estimate of the fake-lepton contributions in the $\mu \mu$ and  regions. The superscript *C* indicates the fake-enriched control region.

The electron fake factor is computed using the fake-enriched region where a same-sign $e\mu$ pair was selected:2In addition to the prompt-lepton contribution, the electron charge-misidentification contribution, denoted by , needs to be subtracted. It is computed using the method described in Sect. 4.3.1. Furthermore, the fake-muon contribution in the $e\mu$ sample is subtracted from the numerator. It is computed as:The fake-muon contribution is not considered in the denominator of the electron fake factor, in Eq. (2), because it is negligible.

The muon fake factor is measured to be $0.14\pm 0.03$, while the electron fake factor is $0.48\pm 0.07$, where the uncertainties are statistical only. A systematic uncertainty of 35% (56%) in the electron (muon) fake factor is estimated from complementary control samples with low jet multiplicity or by applying a different selection to vary the fraction of jets containing heavy-flavour hadrons. The uncertainty in the muon fake factor is larger than in the electron fake factor due lower number of data events available for those checks. The fake-lepton contributions in the nominal region (signal or preselection, denoted collectively by the superscript *R*) are obtained by multiplying the fake factors by the number of events in a region with the same selection as the nominal region, but where at least one lepton is of type :3where the prompt-lepton and the charge misidentifications contributions are subtracted as explained above.

**Fake-lepton contribution estimate for the** $3\ell$ **channel** A method similar to that employed for the $2\ell ^\text {ss}$ channel is applied for the $3\ell$ channel. Here the opposite-sign lepton $\ell _0$ is assumed to be prompt, an assumption that was found to be valid in MC simulation. The fake-enriched region used to calculate the fake factors for the $3\ell$ channel, which is described in Table 3, follows the $3\ell$ preselection conditions except that the jet multiplicity is required to be exactly one. The fake factors for electrons and muons are both calculated by applying a formula analogous to Eq. (1) to the  and  regions, respectively. The muon fake factor is found to be $0.17\pm 0.06$ and the electron fake factor is found to be $0.39\pm 0.07$, where the errors are statistical only. The values are compatible with those obtained for the $2\ell ^\text {ss}$ channel. Additional control samples, defined such that the content is enriched in either $Z$+jets or $t\bar{t}$ events, are used to test the method and to estimate systematic uncertainties of 55% and 81% for the electron and muon fake factors, respectively. The fake-lepton contributions to the nominal regions are then calculated using relations analogous to Eq. (3).

**Fake-lepton contribution estimate for the** $4\ell$ **channel** There are too few data events to apply the fake-factor method in the $4\ell$ channel. Instead, the fake-lepton contribution is estimated from the yields predicted by the MC simulation but corrected using process-dependent scale factors that are extracted in two fake-enriched control regions. The fake-lepton contribution in this channel comes mainly from $t\bar{t}V$ processes, where the fake lepton originates from a *b*-jet. A small component from light quarks is also present. Two data samples designed to contain fake leptons originating from $Z$+jets and $t\bar{t}$ events are used to study the capability of the simulation to describe fake leptons originating from light- and heavy-flavour jets, respectively. The two control samples are labelled Z and T and are defined in Table 3. The samples are required to have three identified leptons. For the Z region, the fake-lepton candidate is assumed not to be part of the lepton pair forming the *Z* boson candidate. For the T region, the fake lepton is assumed to be the lepton with the lower $p_{\mathrm {T}}$ in the same-sign lepton pair. The scale factors are derived independently for fake electrons and fake muons. Four scale factors $\lambda _\mathrm {X}^{\ell }$ (with $\ell =e,\mu$ and $\mathrm {X=Z,T}$) are obtained by solving the system of equations

$$\begin{aligned} N^{\ell }_\mathrm {Data | X}-N^{\ell }_\mathrm {Prompt | X} = \lambda _\text {T}^{\ell }N^{\ell }_{t\bar{t} | \mathrm {X}} + \lambda _\text {Z}^{\ell }N^\ell _{Z+\text {jets} | \mathrm {X}}, \end{aligned}$$

where the event yields $N^{\ell }$ are labelled by the nature of the contribution, data (Data) or simulation (Prompt, $t\bar{t}$ and $Z$+jets), and the equations are derived in each of the respective control region X (Z or T). The obtained scale factors are $\lambda _\text {T}^{e} = 1.12 \pm 0.05$, $\lambda _\text {Z}^{e} = 1.02 \pm 0.07$, $\lambda _\text {T}^{\mu } =1.11 \pm 0.05$ and $\lambda _\text {Z}^{\mu } = 0.94 \pm 0.07$, where the errors are statistical only. Alternative trilepton control samples, where the jet multiplicity and the lepton $p_{\mathrm {T}}$ threshold are varied, are used to estimate a systematic uncertainty of 50% in these scale factors. The scale factors are used as weights to the simulated events that contain a fake lepton according to the fake-lepton flavour and the presence of heavy-flavour jets in the event.

## Signal region optimisation

The hypothetical signal produces four $W^\pm$ bosons in each event. Since at least two leptonic *W* boson decays are needed to lead to the multi-lepton topologies considered in this analysis, all signal events are expected to feature significant $E_{\text {T}}^{\text {miss}}$, while jets are expected from hadronic *W* boson decays for $2\ell ^\text {ss}$ and $3\ell$ channels. Moreover, when the mass of the doubly charged Higgs boson is in the range of 200–300 GeV, each $H^{\pm \pm }$ is produced with a significant momentum and the two subsequent *W* bosons are emitted close to each other in the laboratory frame. Consequently, the two same-sign leptons from the decays of the two *W* bosons tend to be close in the $\eta$–$\phi$ plane. The decay products of the other doubly charged Higgs boson are generally well-separated from the two same-sign leptons.

The analysis channels face different background contributions from the SM. The $2\ell ^\text {ss}$ category is populated with events containing one prompt lepton from a *W* boson, or to a lesser extent from a *Z* boson, and one fake lepton from the hadronic final state produced. The $2\ell ^\text {ss}$ events with two same-sign electrons can also originate from Drell–Yan and $t\bar{t}$ production, where the charge of one of the electrons is misidentified, as explained above. In the $2\ell ^\text {ss}$ and $3\ell$ channels, most of the expected prompt-lepton contribution is due to the production of *WZ* associated with jets, with both bosons subsequently decaying into leptons. This process also produces other features of the signal, such as significant $E_\text {T}^\text {miss}$ and the absence of *b*-jets for most of the production cross-section. For the *WZ* events, the mass of the same-flavour opposite-sign lepton pair is close to the *Z* boson mass, while no such resonant distribution is expected for the signal. In the $4\ell$ channel, the dominant background originates from $t\bar{t}V$ and *ZZ* production. Processes containing top quarks ($t\bar{t}$, $t\bar{t}V$) can lead to events with multiple leptons in the final state. A noticeable feature of those processes is the presence of *b*-jets.

Given these properties of the signal and of the expected background, the following discriminating variables, in addition to $E_{\text {T}}^{\text {miss}}$, are considered:

- $m_{x\ell }$, the invariant mass of the system composed of all selected leptons in the event, where *x* can be 2, 3 or 4.
- $\Delta R_{\ell ^\pm \ell ^\pm }$, the distance in $\eta$–$\phi$ between two same-sign leptons. This variable is used for the $2\ell ^\text {ss}$ and $3\ell$ channels. In the $4\ell$ channel, two such variables can be calculated per event, $\Delta R^\mathrm {min}_{\ell ^\pm \ell ^\pm }$ and $\Delta R^\mathrm {max}_{\ell ^\pm \ell ^\pm }$, denoting the minimum and maximum values, respectively.
- $m_\mathrm {jets}$, the invariant mass of the system composed of all jets in the event. When there are more than four jets in the event, only the leading four jets are used. This variable is used only for the $2\ell ^\text {ss}$ channel.
- $p_{\mathrm {T}} ^{\text {leading jet}}$, the transverse momentum of the highest-$p_{\mathrm {T}}$ jet.
- $\Delta \phi ({\ell \ell ,E_{\text {T}}^{\text {miss}}})$, the difference in azimuth between the dilepton system and $E_{\text {T}}^{\text {miss}}$. This variable is used in the $2\ell ^\text {ss}$ channel.
- $\Delta R_{\ell -\mathrm {jet}}$, the minimal distance in $\eta$–$\phi$ between any lepton and its closest jet. This variable is used in the $3\ell$ channel.
- *S*, is a variable used for the $2\ell ^\text {ss}$ channel to describe the event topology in the transverse plane, and defined using the spread of the $\phi$ angles of the leptons, $E_{\text {T}}^{\text {miss}}$, and jets as follows:
  $$\begin{aligned} S = \frac{{\mathcal {R} }(\phi _{\ell _1},\phi _{\ell _2},\phi _{E_{\text {T}}^{\text {miss}}})\cdot {\mathcal {R} }(\phi _{j1},\phi _{j2},\ldots )}{{\mathcal {R} }(\phi _{\ell _1,},\phi _{\ell _2},\phi _{E_{\text {T}}^{\text {miss}}},\phi _{j1},\phi _{j2},\ldots ) }, \end{aligned}$$
  where the $\mathcal {R}$ is the root mean square that quantifies the spread, ${\mathcal {R}} (\phi _1, \ldots , \phi _n ) = \sqrt{\frac{1}{n} \sum \limits _{i=1}^{n} (\phi _i - \overline{\phi } )^2}$. The azimuthal angles $\phi$ are bounded in $(-\pi , \pi ]$, and the bound is considered in the calculation. The *S* variable is expected to be on average smaller for the signal than for the background for low $H^{\pm \pm }$ mass values.

**Fig. 1:**
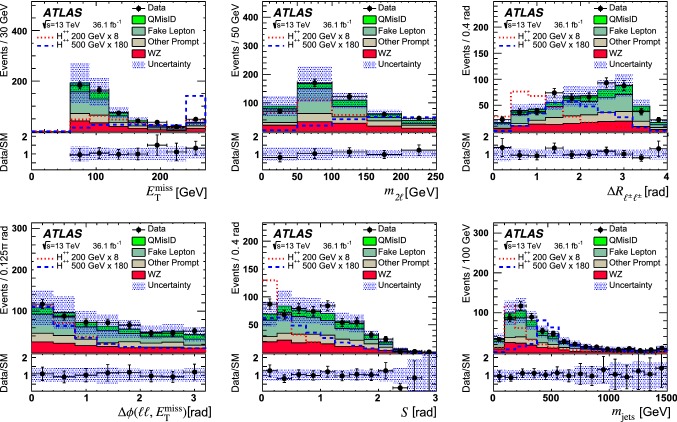
Distribution of variables used for the signal region optimisation of the $2\ell ^\text {ss}$ final state. The events are selected with the preselection requirements listed in Table 2. The data (dots) are compared with the predictions (histograms) that include the contributions from the dominant prompt-lepton background (*WZ*), other prompt-lepton backgrounds, processes where a fake lepton is reconstructed, and electrons with misidentified charge (QMisID). The expected signal distributions corresponding to two $H^{\pm \pm }$ masses are also shown, scaled up for visibility. The last bin includes overflows. In each figure the bottom panel shows the ratio of data to the prediction, where the band around unity represents the total uncertainty of the SM prediction

**Fig. 2:**
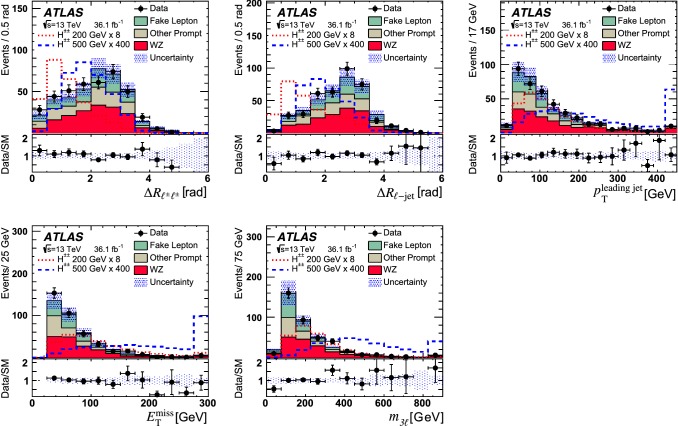
Distribution of variables used for the signal region optimisation of the $3\ell$ channel (a detailed description can be found in the caption of Fig. 1)

**Fig. 3:**
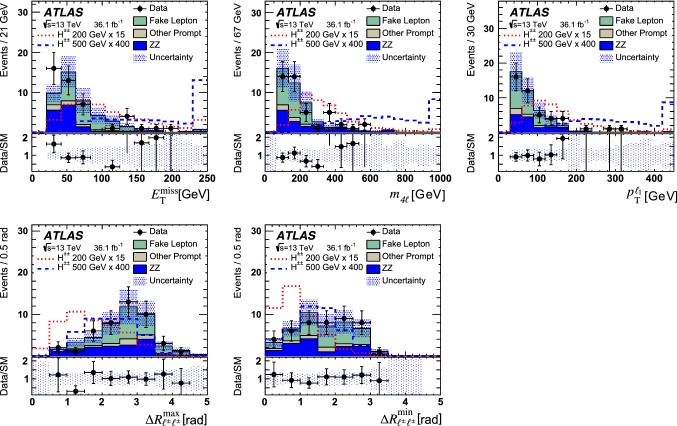
Distribution of variables used for the signal region optimisation of the $4\ell$ channel (a detailed description can be found in the caption of Fig. 1)

The distributions of the selected variables for the $2\ell ^\text {ss}$, $3\ell$ and $4\ell$ channels are shown at preselection level in Figs. 1, 2 and 3, respectively. The data are compared with the sum of the prompt lepton, fake lepton and charge-misidentified lepton background predictions. The prompt-lepton backgrounds are estimated with simulations while the background from fake leptons and charge-flipped leptons are measured with the methods described in the previous section. Good agreement is observed in both normalisation and shape, demonstrating that the background contributions are well modelled. The expected signal distributions for various $H^{\pm \pm }$ masses are also shown to illustrate the discriminating power of the selected variables.

The strategy used to extract the signal is based on rectangular cut optimisation using the TMVA package [59]. For each $m_{H^{\pm \pm }}$ hypothesis, six signal regions are defined using the following lepton flavour content: in the $2\ell ^\text {ss}$ channel, three signal regions are optimised separately for *ee*, $e\mu$ and $\mu \mu$ channels; in the $3\ell$ channel, the signal regions are optimised separately for events with no same-flavour opposite-sign lepton pairs (SFOS 0, for which the SM background is small) and for events with one or two such pairs (SFOS 1,2); the $4\ell$ channel is treated globally, with no further lepton flavour distinction. The selection criteria used to define the signal regions are shown in Table 4. The optimisation is performed as a function of the $H^{\pm \pm }$ mass for $m_{H^{\pm \pm }}=200, 300, 400$ and 500 GeV, and seeks the best expected signal significance. The last optimisation point is applied to $m_{H^{\pm \pm }} \ge 600$ GeVas well, since the signal discrimination power does not vary significantly in this regime.

In order to verify the background estimate reliability for the signal region, three further checks were performed: the optimised cuts were applied individually, the cuts were applied successively, or each cut was inverted while the other cuts were applied. The agreement between data and prediction remains adequate for all those cases.

**Table 4:** The selection criteria used to define the signal regions

	$2\ell ^\text {ss}$			$3\ell$		$4\ell$
Selection criteria	$e^\pm e^\pm$	$e^\pm \mu ^\pm$	$\mu ^\pm \mu ^\pm$	SFOS 0	SFOS 1,2	
$m_{H^{\pm \pm }}=200$ GeV						
$$E_{\text {T}}^{\text {miss}} $$ [GeV]	$$>100$$	$$>100$$	$$>100$$	$$ > 45$$	$$> 45$$	$$>60$$
$m_{x\ell }$ [GeV]	[25, 130]	[15, 150]	[35, 150]	$> 160$	$> 170$	$>230$
$\Delta R_{\ell ^{\pm }\ell ^{\pm }}$[rad.]	$<0.8$	$<1.8$	$<0.9$	[0.15, 1.57]	[0.00, 1.52]	
$\Delta \phi (\ell \ell ,E_{\text {T}}^{\text {miss}})$ [rad.]	$<1.1$	$<1.3$	$<1.3$			
*S* [rad.]	$$<0.3$$	$$<0.3$$	$$<0.2 $$			
$m_\mathrm {jets}$ [GeV]	[140, 770]	[95, 330]	[95, 640]			
$\Delta R_{\ell -\mathrm {jet}}$ [rad.]				[0.08, 1.88]	[0.07, 1.31]	
$$ p_{\mathrm {T}} ^{\text {leading jet}}$$ [GeV]				$$> 80 $$	$$ > 55$$	
$$ p_{\mathrm {T}} ^{\ell _1}$$ [GeV]						$$ > 65$$
$\Delta R^\text {min}_{\ell ^{\pm }\ell ^{\pm }}$ [rad.]						[0.16, 1.21]
$\Delta R^\text {max}_{\ell ^{\pm }\ell ^{\pm }}$ [rad.]						[0.27, 2.03]
$m_{H^{\pm \pm }}=300$ GeV						
$$E_{\text {T}}^{\text {miss}} $$ [GeV]	$$>200$$	$$>200$$	$$>200$$	$$ > 65$$	$$> 55$$	$$>60$$
$m_{x\ell }$ [GeV]	[105, 340]	[80, 320]	[80, 320]	$> 170$	$> 210$	$>270$
$\Delta R_{\ell ^{\pm }\ell ^{\pm }}$ [rad.]	$<1.4$	$<1.8$	$<1.8$	[0.18, 2.23]	[0.08, 2.23]	
$\Delta \phi (\ell \ell ,E_{\text {T}}^{\text {miss}})$ [rad.]	$<2.1$	$<2.4$	$<2.4$			
*S* [rad.]	$$<0.4$$	$$<0.4$$	$$<0.4 $$			
$m_\mathrm {jets}$ [GeV]	[180, 770]	[130, 640]	[130, 640]			
$\Delta R_{\ell j}$ [rad.]				[0.27, 2.37]	[0.21, 2.08]	
$$ p_{\mathrm {T}} ^{\text {leading jet}}$$ [GeV]				$$> 95 $$	$$ > 80$$	
$$ p_{\mathrm {T}} ^{\ell _1}$$ [GeV]						$$ > 45$$
$\Delta R^\text {min}_{\ell ^{\pm }\ell ^{\pm }}$ [rad.]						[0.09, 1.97]
$\Delta R^\text {max}_{\ell ^{\pm }\ell ^{\pm }}$ [rad.]						[0.44, 2.68]
$m_{H^{\pm \pm }}=400$ GeV						
$$E_{\text {T}}^{\text {miss}} $$ [GeV]	$$>200$$	$$>200$$	$$>200$$	$$ > 65$$	$$> 85$$	$$>60$$
$m_{x\ell }$ [GeV]	[105, 340]	[80, 350]	[80, 350]	$> 230$	$> 250$	$>270$
$\Delta R_{\ell ^{\pm }\ell ^{\pm }}$ [rad.]	$<2.2$	$<1.8$	$<1.8$	[0.22, 2.39]	[0.29, 2.69]	
$\Delta \phi (\ell \ell ,E_{\text {T}}^{\text {miss}})$ [rad.]	$<2.4$	$<2.4$	$<2.4$			
*S* [rad.]	$$<0.6$$	$$<0.6$$	$$<0.5 $$			
$m_\mathrm {jets}$ [GeV]	[280, 1200]	[220, 1200]	[220, 1200]			
$\Delta R_{\ell j}$ [rad.]				[0.30, 2.59]	[0.31, 2.30]	
$$ p_{\mathrm {T}} ^{\text {leading jet}}$$ [GeV]				$$> 120 $$	$$ > 100$$	
$$ p_{\mathrm {T}} ^{\ell _1}$$ [GeV]						$$ > 110$$
$\Delta R^\text {min}_{\ell ^{\pm }\ell ^{\pm }}$ [rad.]						[0.39, 2.22]
$\Delta R^\text {max}_{\ell ^{\pm }\ell ^{\pm }}$ [rad.]						[0.55, 2.90]
$m_{H^{\pm \pm }}=500$–700 GeV						
$$E_{\text {T}}^{\text {miss}} $$ [GeV]	$$>250$$	$$>250$$	$$>250$$	$$ > 120$$	$$> 100$$	$$>60$$
$m_{x\ell }$ [GeV]	[105, 730]	[110, 440]	[110, 440]	$> 230$	$> 300$	$>370$
$\Delta R_{\ell ^{\pm }\ell ^{\pm }}$ [rad.]	$<2.6$	$<2.2$	$<2.2$	[0.39, 3.11]	[0.29, 2.85]	
$\Delta \phi (\ell \ell ,E_{\text {T}}^{\text {miss}})$ [rad.]	$<2.6$	$<2.4$	$<2.4$			
*S* [rad.]	$$<1.1$$	$$<1.1$$	$$<1.1 $$			
$m_\mathrm {jets}$ [GeV]	$>440$	$>470$	$>470$			
$\Delta R_{\ell j}$ [rad.]				[0.60, 2.68]	[0.31, 2.53]	
$$ p_{\mathrm {T}} ^{\text {leading jet}}$$ [GeV]				$$> 130 $$	$$ > 130$$	
$$ p_{\mathrm {T}} ^{\ell _1}$$ [GeV]						$$ > 160$$
$\Delta R^\text {min}_{\ell ^{\pm }\ell ^{\pm }}$ [rad.]						[0.53, 3.24]
$\Delta R^\text {max}_{\ell ^{\pm }\ell ^{\pm }}$ [rad.]						[0.59, 2.94]

The variables are described in Sect. 5

## Systematic uncertainties

The theoretical uncertainties associated with the signal prediction originate from the PDFs, the matrix element calculation and the parton shower simulation. The uncertainties related to PDFs are evaluated using the Hessian method provided in LHAPDF6 [60] and are found to be in the range from 2.5% to 4.5%. The uncertainty of the parton shower simulation is assessed by comparing PYTHIA (with A14 tune) and Herwig++ (with UEEE5 tune [61]), and is found to be 2.4%, 1.7%, and 3.8% for the $2\ell ^\text {ss}$, $3\ell$, and $4\ell$ channels, respectively. The higher-order corrections are assumed to induce an additional 15% uncertainty in the cross-section calculations [33]. Combining those uncertainties in quadrature, an overall uncertainty of 17% is obtained for the signal normalisation.

The theoretical uncertainties associated with the largest SM backgrounds, *VV* [48] (including same-sign *WWqq* and *WZ* processes) and $t\overline{t}V$ [62], are estimated using dedicated MC samples, where the factorisation and renormalisation scales are varied independently by factors of 2 and 0.5 and the parton shower parameters are varied within the given model uncertainties. The theoretical uncertainties obtained are 24% for *VV* and 17% for $t\overline{t}V$. The uncertainties related to PDFs are found to be negligible. The uncertainty associated with the $V\gamma$ contributions is taken to be 25%, as indicated by dedicated studies using converted photons. The uncertainties related to the *VVV* and *tZ* process predictions are taken from the respective inclusive cross-section measurements [63, 64]. For other rare backgrounds which have no dedicated measurements yet ($tt\bar{t}$, $t\bar{t}W^+W^-$), uncertainties of 50% are assumed and are found to have a negligible impact on the sensitivity.

The experimental uncertainties arise from the accuracy of the detector simulation and from the uncertainties associated with the data-driven methods that are used to estimate the instrumental backgrounds. These uncertainties originate from the following sources:

- The uncertainties related to event reconstruction include the lepton [52, 65] and the jet [66] energy scales and resolutions and the uncertainties in the reconstruction of $E_{\text {T}}^{\text {miss}}$  [58]. The impact of this type of uncertainty on the signal and background yields is in the range 3–$8\%$ and 10–$30\%$, respectively.
- The uncertainties related to the efficiencies of electron [51] and muon [52] reconstruction and identification, including the uncertainties in the trigger efficiency are estimated in dedicated studies. The impact of this type of uncertainty on the signal and background yields is found to be in the range 4–$6\%$ and 2–$5\%$, respectively. The uncertainties related to the *b*-jet identification algorithms, used in the analysis to veto events containing *b*-jets, are found to be negligible.
- The uncertainties originating from data-taking conditions include the luminosity measurement and the pile-up simulation procedure. The uncertainty of the integrated luminosity is $2.1\%$, determined using a methodology similar to that detailed in Ref. [67]. The uncertainty from the pile-up simulation is about $5\%$.
- The uncertainties related to the background contributions from electron charge misidentification are 22–$28\%$ for the $2\ell ^\text {ss}$ *ee* and $e\mu$ channels. The uncertainties of the fake-lepton contributions range from 50 to 250%, and mainly originate from the fake factors ($2\ell ^\text {ss}$ and $3\ell$ channels) and the scale factors for MC simulation ($4\ell$ channel) described in Sect. 4.3.2 and the statistics of the control samples. The uncertainties exceed $100\%$ in some cases due to the subtraction of the prompt-lepton contributions in the fake-lepton control regions.

The theoretical and experimental systematic uncertainties described above are assumed to be correlated amongst the various signal regions in the interpretation of the final results. Overall, the sensitivity of the search is dominated by the statistical uncertainty of the event yield in the signal regions.

## Results

**Fig. 4:**
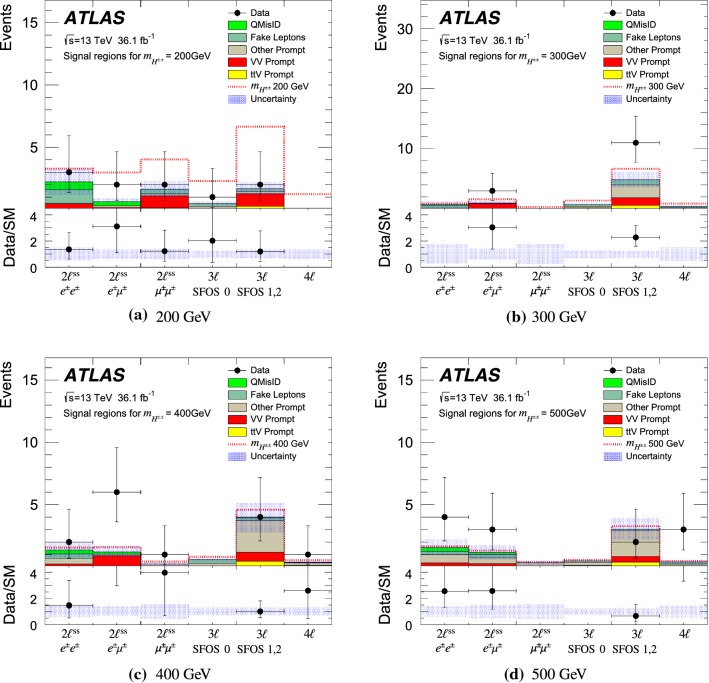
Event yields in the signal regions optimised for the $m_{H^{\pm \pm }}$= 200, 300, 400 and 500 GeV searches. The bottom panel shows the ratio of the data to the total background prediction, where the band illustrates the total uncertainty of the SM background. The error bars attributed to data are estimated assuming a Poisson distribution with the average equal to the respective yields. The signal prediction is represented as a dotted histogram, stacked on the SM background

The expected and observed event yields in the signal regions are shown in Fig. 4 and Table 5. For a $H^{\pm \pm }$ mass of 200 GeV, substantial signal yield is expected in all channels, and the analysis sensitivity is found to be comparable across the $2\ell ^\text {ss}$, $3\ell$ and $4\ell$ channels. No significant excess has been observed. Table 5 also includes the overall signal acceptance *A*, defined as the number of selected events selected in a given channel divided by the total number of $pp \rightarrow H^{\pm \pm }H^{\mp \mp }\rightarrow W^\pm W^\pm W^\mp W^\mp$ events and representing the signal reduction due to phase space acceptance, branching ratio and detector efficiency.

The statistical analysis of the results is based on a likelihood ratio test [68] using the CL$_\mathrm {s}$ method [69]. The parameter of interest is the signal strength, defined as the cross-section of the hypothetical contribution from physics beyond the SM in units of the cross-section of the benchmark model. The likelihood function is constructed from Poisson probability distributions of counting experiments for each of the six channels in each signal region. The systematic uncertainties are treated as nuisance parameters implemented in the likelihood functions with Gaussian constraints.

**Fig. 5:**
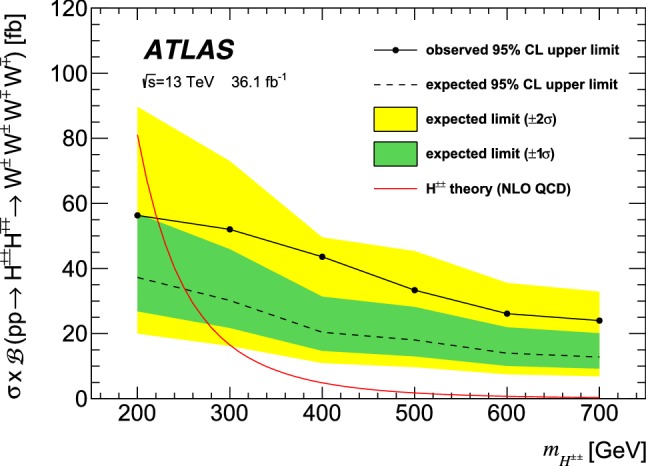
Observed and expected upper limits for $pp \rightarrow H^{\pm \pm }H^{\mp \mp }\rightarrow W^\pm W^\pm W^\mp W^\mp$ cross-section times branching fraction at $95\%$ CL obtained from the combination of $2\ell ^\text {ss}$, $3\ell$ and $4\ell$ channels. The region above the observed limit is excluded by the measurement. The bands represent the expected exclusion curves within one and two standard deviations. The theoretical prediction [3] including the NLO QCD corrections [33] is also shown and is excluded for $m_{H^{\pm \pm }}$ < 220 GeV

The expected and observed upper limits of the $H^{\pm \pm }\rightarrow W^\pm W^\pm$ cross-section at 95% confidence level (CL), obtained from the combination of $2\ell ^\text {ss}$, $3\ell$ and $4\ell$ channels for the six $H^{\pm \pm }$ mass hypotheses are shown in Fig. 5. Assuming a linear interpolation of the sensitivity between neighbouring mass hypotheses, and the cross-section of the benchmark model, the observed (expected) lower limit on the mass of the $H^{\pm \pm }$ boson is 220 GeV (250 GeV) at 95% CL.

**Table 5:** Event yields in the signal regions of corresponding targeted masses of $H^{\pm \pm }$

	$2\ell ^\text {ss}$			$3\ell$		$4\ell$
Subchannel	$e^\pm e^\pm$	$e^\pm \mu ^\pm$	$\mu ^\pm \mu ^\pm$	SFOS 0	SFOS 1,2	
$m_{H^{\pm \pm }}=200$ GeV						
Prompt lepton	$0.5 \pm 0.2$	$0.3\pm 0.2$	$1.3\pm 0.6$	$0.3\pm 0.1$	$1.4 \pm 0.5$	$0.07 \pm 0.03$
QMisID	$0.6 \pm 0.2$	$0.4\pm 0.1$	−	−	−	−
Fake lepton	$$1 \pm 1 $$	$$ < 0.4$$	$$0.4\pm 0.3$$	$$0.2\pm 0.1$$	$$0.2\pm 0.1$$	$$0.03 \pm 0.02$$
Total background	$2\pm 1$	$0.6 \pm 0.3$	$1.7\pm 0.7$	$0.5\pm 0.1$	$1.7\pm 0.6$	$0.11\pm 0.05$
Signal	$1.1 \pm 0.2$	$2.3\pm 0.4$	$2.4\pm 0.4$	$1.8\pm 0.3$	$5.0\pm 0.9$	$1.1\pm 0.2$
*A* [%]	0.037	0.080	0.082	0.061	0.17	0.038
$n_{95}$	12.3	7.1	7.5	4.1	7.7	3.8
Data	3	2	2	1	2	0
$m_{H^{\pm \pm }}=300$ GeV						
Prompt lepton	$0.1\pm 0.1$	$0.9\pm 0.4$	$0.02\pm 0.02$	$0.4\pm 0.1$	$4\pm 1$	$0.3 \pm 0.1$
QMisID	$0.1\pm 0.1$	$0.07\pm 0.04$	−	−	−	−
Fake lepton	$0.4\pm 0.5$	$<0.2$	$<0.4$	$0.3\pm 0.2$	$0.8\pm 0.4$	$0.2 \pm 0.2$
Total background	$0.7\pm 0.5$	$1.0\pm 0.5$	$0.02\pm 0.02$	$0.8\pm 0.2$	$5\pm 2$	$0.5\pm 0.2$
Signal	$0.16\pm 0.03$	$0.6\pm 0.1$	$0.29\pm 0.05$	$0.6\pm 0.1$	$1.8\pm 0.3$	$0.43\pm 0.08$
*A* [%]	0.027	0.10	0.049	0.11	0.30	0.071
$n_{95}$	4.0	9.6	3.0	3.1	22.7	3.8
Data	0	3	0	0	11	0
$m_{H^{\pm \pm }}=400$ GeV						
Prompt lepton	$0.7\pm 0.3$	$1.0\pm 0.4$	$0.2\pm 0.1$	$0.3\pm 0.1$	$4\pm 1$	$0.3 \pm 0.1$
QMisID	$0.3\pm 0.1$	$0.2\pm 0.1$	−	−	−	−
Fake lepton	$0.4\pm 0.5$	$<0.3$	$<0.4$	$0.3\pm 0.2$	$0.2\pm 0.1$	$0.05\pm 0.04$
Total background	$1.4\pm 0.6$	$1.2\pm 0.5$	$0.3\pm 0.1$	$0.6\pm 0.2$	$4\pm 1$	$0.4\pm 0.1$
Signal	$0.20\pm 0.04$	$0.38\pm 0.07$	$0.19\pm 0.03$	$0.23\pm 0.04$	$0.6\pm 0.1$	$0.17\pm 0.03$
*A* [%]	0.11	0.21	0.11	0.13	0.36	0.092
$n_{95}$	10.4	18.3	6.4	3.1	10.4	4.3
Data	2	6	1	0	4	1
$m_{H^{\pm \pm }}=500$ GeV						
Prompt lepton	$1.0\pm 0.4$	$0.7\pm 0.3$	$0.3\pm 0.2$	$0.4\pm 0.1$	$3\pm 1$	$0.2\pm 0.1$
QMisID	$0.3\pm 0.1$	$0.2\pm 0.1$	−	−	−	−
Fake lepton	$$0.2\pm 0.5$$	$$0.3\pm 0.5$$	$$ <0.4$$	$$ 0.11\pm 0.06$$	$$0.10\pm 0.05$$	$$0.2\pm 0.2$$
Total background	$1.6\pm 0.6$	$1.2\pm 0.6$	$0.3\pm 0.2$	$0.5\pm 0.1$	$3.0\pm 0.8$	$0.4\pm 0.2$
Signal	$0.10\pm 0.02$	$0.16\pm 0.03$	$0.07\pm 0.01$	$0.09\pm 0.02$	$0.24\pm 0.04$	$0.06\pm 0.01$
*A* [%]	0.16	0.25	0.11	0.14	0.37	0.098
*A* [%] $m_{H^{\pm \pm }}=600$ GeV	0.22	0.36	0.16	0.17	0.44	0.11
*A* [%] $m_{H^{\pm \pm }}=700$ GeV	0.26	0.38	0.17	0.19	0.48	0.12
$n_{95}$	8.6	12.7	3.8	3.0	7.9	4.9
Data	4	3	0	0	2	3

The signal yield is for the corresponding mass point and is normalised to the luminosity of $36.1\, \hbox {fb}^{-1}$. The dominant background from prompt-lepton sources is from the *WZ* process in the $2\ell ^\text {ss}$ channel. For the $3\ell$ and $4\ell$ channels, the dominant background from prompt-lepton sources is from *WZ* and $t\overline{t}V$ processes. The overall signal acceptance *A* and the upper limit of extra contribution to each signal region at 95% confidence level $n_{95}$ are also presented. The data and SM prediction yields obtained for $m_{H^{\pm \pm }}=500$ GeV are also valid for $m_{H^{\pm \pm }}=600$ and 700 GeV

## Conclusion

A search for the pair production of doubly charged Higgs scalar bosons with subsequent decays into *W* bosons is performed in proton–proton collisions at a centre-of-mass energy of 13 TeV. The data sample was collected by the ATLAS experiment at the LHC and corresponds to an integrated luminosity of $36.1\, \hbox {fb}^{-1}$. The search for the $H^{\pm \pm } \rightarrow W^{\pm } W^{\pm }$ decay mode, not considered in previous analyses at colliders, is motivated by a model with an extended scalar sector that includes a triplet in addition to the Standard Model scalar doublet. The analysis proceeds through the selection of multi-lepton events in three channels (a pair of same-sign leptons, three leptons and four leptons) with missing transverse momentum and jets. The signal region is optimised as a function of the $H^{\pm \pm }$ mass. The data are found to be in good agreement with the Standard Model predictions for all channels investigated. Combining those channels, the model considered is excluded at 95% confidence level for $H^{\pm \pm }$ boson masses between 200 and 220 GeV.

## Footnotes

1 ATLAS uses a right-handed coordinate system with its origin at the nominal interaction point (IP) in the centre of the detector and the *z*-axis along the beam pipe. The *x*-axis points from the IP to the centre of the LHC ring, and the *y*-axis points upwards. Cylindrical coordinates $(r,\phi )$ are used in the transverse plane, $\phi$ being the azimuthal angle around the *z*-axis. The pseudorapidity is defined in terms of the polar angle $\theta$ as $\eta = -\ln \tan (\theta /2)$. Angular distance is measured in units of $\Delta R \equiv \sqrt{(\Delta \eta )^{2} + (\Delta \phi )^{2}}$.

## References

[1] Schechter J, Valle JWF (1980). Neutrino masses in $$SU(2) \otimes U(1)$$ theories. Phys. Rev. D.

[2] Fileviez Perez P, Han T, Huang G-y, Li T, Wang K (2008). Neutrino masses and the CERN LHC: Testing the type II seesaw mechanisim. Phys. Rev. D.

[3] A. Arhrib et al., The Higgs potential in the type II seesaw model. Phys. Rev. D 84, 095005 (2011) (We would like to thank Gilbert Moultaka for the implementation in CalcHEP of the doublet-triplet-model and helpful discussions on the phenomenological aspects of the analysis). arXiv: 1105.1925 [hep-ph]

[4] Kanemura S, Kikuchi M, Yagyu K, Yokoya H (2014). Bounds on the mass of doubly-charged Higgs bosons in the same-sign diboson decay scenario. Phys. Rev. D.

[5] Kang Z, Li J, Li T, Liu Y, Ning G-Z (2015). Light doubly charged Higgs boson via the $$WW^{*}$$ channel at LHC. Eur. Phys. J. C.

[6] OPAL collaboration, Search for doubly charged Higgs bosons with the OPAL detector at LEP. Phys. Lett. B **526**, 221 (2002). arXiv: hep-ex/0111059

[7] H1 Collaboration, Search for doubly-charged Higgs boson production at HERA. Phys. Lett. B **638**, 432 (2006). arXiv: hep-ex/0604027

[8] CDF Collaboration, Search for new physics in high $$p_{T}$$ like-sign Dilepton events at CDF II. Phys. Rev. Lett. **107**, 181801 (2011). arXiv: 1108.0101 [hep-ex] 10.1103/PhysRevLett.107.181801 22107622

[9] ATLAS Collaboration, Search for doubly-charged Higgs bosons in like-sign dilepton final states at $$\sqrt{s} = 7\, TeV$$ with the ATLAS detector. Eur. Phys. J. C **72**, 2244 (2012). arXiv: 1210.5070 [hep-ex]

[10] CMS Collaboration, A search for a doubly-charged Higgs boson in pp collisions at $$\sqrt{s} = 7\, TeV$$. Eur. Phys. J. C **72**, 2189 (2012). arXiv: 1207.2666 [hep-ex]

[11] ATLAS Collaboration, Search for doubly charged Higgs boson production in multi-lepton final states with the ATLAS detector using proton–proton collisions at $$\sqrt{s} = 13\, TeV$$. Eur. Phys. J. C **78**, 199 (2018). arXiv: 1710.09748 [hep-ex] 10.1140/epjc/s10052-018-5661-z PMC6560737 31265007

[12] CMS Collaboration, Study of vector boson scattering and search for new physics in events with two same-sign leptons and two jets. Phys. Rev. Lett. **114**, 051801 (2015). arXiv: 1410.6315 [hep-ex] 10.1103/PhysRevLett.114.051801 25699433

[13] CMS Collaboration, Observation of electroweak production of same-sign W boson pairs in the two jet and two same-sign lepton final state in proton-proton collisions at $$\sqrt{s} = 13\, TeV$$. Phys. Rev. Lett. **120**, 081801 (2018). arXiv: 1709.05822 [hep-ex] 10.1103/PhysRevLett.120.081801 29542998

[14] Georgi H, Machacek M (1985). Doubly charged Higgs bosons. Nucl. Phys..

[15] Englert C, Re E, Spannowsky M (2013). Triplet Higgs boson collider phenomenology after the LHC. Phys. Rev..

[16] ATLAS Collaboration, Search for Higgs boson decays to beyond-the-Standard-Model light bosons in four-lepton events with the ATLAS detector at $$\sqrt{s} = 13\, TeV$$ (2018). arXiv: 1802.03388 [hep-ex]

[17] ATLAS Collaboration, Search for electroweak production of supersymmetric particles in final states with two or three leptons at $$\sqrt{s} = 13\, TeV$$ with the ATLAS detector. (2018). arXiv: 1803.02762 [hep-ex] 10.1140/epjc/s10052-018-6423-7 PMC6383936 30872954

[18] ATLAS Collaboration, Search for supersymmetry in final states with two same-sign or three leptons and jets using $$36\, fb^{-1}$$ of $$\sqrt{s} = 13\, TeV$$ pp collision data with the ATLAS detector. JHEP **09**, 084 (2017). arXiv: 1706.03731 [hep-ex]

[19] ATLAS Collaboration, The ATLAS experiment at the CERN large Hadron Collider. JINST **3**, S08003 (2008)

[20] ATLAS Collaboration, ATLAS Insertable B-Layer Technical Design Report, ATLAS-TDR-19 (2010). https://cds.cern.ch/record/1291633. ATLAS Insertable B-Layer Technical Design Report Addendum, ATLAS-TDR-19-ADD-1 (2012). https://cds.cern.ch/record/1451888

[21] ATLAS Collaboration, Performance of the ATLAS trigger system in 2015. Eur. Phys. J. C **77**, 317 (2017). arXiv: 1611.09661 [hep-ex] 10.1140/epjc/s10052-017-4852-3 PMC5586243 28943784

[22] ATLAS Collaboration, The ATLAS simulation infrastructure. Eur. Phys. J. C **70**, 823 (2010). arXiv: 1005.4568 [physics.ins-det]

[23] Agostinelli S (2003). Geant4: a simulation toolkit. Nucl. Instrum. Meth. Phys. Res. A.

[24] ATLAS Collaboration, The simulation principle and performance of the ATLAS fast calorimeter simulation FastCaloSim. ATL-PHYS-PUB-2010-013 (2010). https://cds.cern.ch/record/1300517

[25] T. Sjöstrand, S. Mrenna, P. Z. Skands, PYTHIA 6.4 physics and manual. JHEP **05**, 026 (2006). arXiv: hep-ph/0603175

[26] T. Sjöstrand, S. Mrenna, P. Z. Skands, A brief introduction to PYTHIA 8.1. Comput. Phys. Commun. **178**, 852 (2008). arXiv: 0710.3820 [hep-ph]

[27] ATLAS Collaboration, Monte Carlo Generators for the Production of a W or $$Z/\gamma ^{*}$$ Boson in Association with Jets at ATLAS in Run 2. ATL-PHYS-PUB-2016-003. (2015). https://cds.cern.ch/record/2120133

[28] Martin A, Stirling WJ, Thorne RS, Watt G (2009). Parton distributions for the LHC. Eur. Phys. J. C.

[29] Belyaev A, Christensen ND, Pukhov A (2013). CalcHEP 3.4 for collider physics within and beyond the Standard Model. Comput. Phys. Commun..

[30] Pumplin J (2002). New generation of parton distributions with uncertainties from global QCD analysis. JHEP.

[31] Nadolsky PM (2008). Implications of CTEQ global analysis for collider observables. Phys. Rev. D.

[32] ATLAS Collaboration, ATLAS Pythia 8 tunes to 7 TeV data. ATL-PHYS-PUB-2014-021 (2014). https://cds.cern.ch/record/1966419

[33] Muhlleitner M, Spira M (2003). A Note on doubly charged Higgs pair production at hadron colliders. Phys. Rev. D.

[34] Lange DJ (2001). The EvtGen particle decay simulation package. Nucl. Instrum. Meth. A.

[35] T. Gleisberg et al., Event generation with SHERPA 1.1. JHEP **02**, 007 (2009). arXiv: 0811.4622 [hep-ph]

[36] Lai H-L (2010). New parton distributions for collider physics. Phys. Rev. D.

[37] Alwall J (2014). The automated computation of tree-level and next-to-leading order differential cross sections, and their matching to parton shower simulations. JHEP.

[38] Ball RD (2015). Parton distributions for the LHC Run II. JHEP.

[39] Bahr M (2008). Herwig++ physics and manual. Eur. Phys. J. C.

[40] Seymour MH, Siodmok A (2013). Constraining MPI models using $$\sigma _{eff}$$ and recent Tevatron and LHC Underlying Event data. JHEP.

[41] Skands P (2010). Tuning Monte Carlo generators: The Perugia tunes. Phys. Rev. D.

[42] Re E (2011). Single-top Wt-channel production matched with parton showers using the POWHEG method. Eur. Phys. J. C.

[43] Alioli S, Nason P, Oleari C, Re E (2009). NLO single-top production matched with shower in POWHEG: s- and t-channel contributions. JHEP.

[44] Grazzini M, Kallweit S, Rathlev D, Wiesemann M (2016). $$W^{\pm }Z$$ production at hadron colliders in NNLO QCD. Phys. Lett. B.

[45] Caola F, Melnikov K, Rontsch R, Tancredi L (2015). QCD corrections to ZZ production in gluon fusion at the LHC. Phys. Rev. D.

[46] Grazzini M, Kallweit S, Rathlev D (2015). ZZ production at the LHC: Fiducial cross sections and distributions in NNLO QCD. Phys. Lett. B.

[47] ATLAS Collaboration, Modelling of the $$t\bar{t}H$$ and $$t\bar{t}V(V = W, Z)$$ processes for $$\sqrt{s}= 13\, TeV$$ ATLAS analyses, ATL-PHYS-PUB-2016-005 (2016). https://cds.cern.ch/record/2120826

[48] ATLAS Collaboration, Multi-Boson Simulation for 13 TeV ATLAS Analyses, ATL-PHYS-PUB-2017-005 (2017). https://cds.cern.ch/record/2261933

[49] ATLAS Collaboration, Validation of Monte Carlo event generators in the ATLAS Collaboration for LHC Run 2, ATL-PHYS-PUB-2016-001. (2016). https://cds.cern.ch/record/2119984

[50] ATLAS Collaboration, Vertex Reconstruction Performance of the ATLAS Detector at $$\sqrt{s} = 13\, TeV$$, ATL-PHYS-PUB-2015-026 (2015). https://cds.cern.ch/record/2037717

[51] ATLAS Collaboration, Electron efficiency measurements with the ATLAS detector using the 2015 LHC proton-proton collision data, ATLAS-CONF-2016-024 (2016). https://cds.cern.ch/record/2157687 10.1140/epjc/s10052-017-4756-2 PMC5434979 28579919

[52] ATLAS Collaboration, Muon reconstruction performance of the ATLAS detector in proton–proton collision data at $$\sqrt{s} = 13\, TeV$$. Eur. Phys. J. C **76**, 292 (2016). arXiv: 1603.05598 [hep-ex] 10.1140/epjc/s10052-016-4120-y PMC5321258 28280436

[53] ATLAS Collaboration, Topological cell clustering in the ATLAS calorimeters and its performance in LHC Run 1. Eur. Phys. J. C **77**, 490 (2017). arXiv: 1603.02934 [hep-ex] 10.1140/epjc/s10052-017-5004-5 PMC5586976 28943797

[54] Cacciari M, Salam GP, Soyez G (2008). The anti-$$k_{t}$$ jet clustering algorithm. JHEP.

[55] Cacciari M, Salam GP, Soyez G (2012). FastJet user manual. Eur. Phys. J. C.

[56] ATLAS Collaboration, Performance of pile-up mitigation techniques for jets in pp collisions at $$\sqrt{s} = 8\, TeV$$ using the ATLAS detector. Eur. Phys. J. C **76**, 581 (2016). arXiv: 1510.03823 [hep-ex] 10.1140/epjc/s10052-016-4395-z PMC5335592 28316490

[57] ATLAS Collaboration, Performance of b-jet identification in the ATLAS experiment. JINST **11**, P04008 (2016). arXiv: 1512.01094 [hep-ex]

[58] ATLAS Collaboration, Performance of missing transverse momentum reconstruction with the ATLAS detector using proton–proton collisions at $$\sqrt{s} = 13\, TeV$$, (2018), arXiv: 1802.08168 [hep-ex] 10.1140/epjc/s10052-018-6288-9 PMC6394290 30880822

[59] A. Hoecker et al., TMVA: Toolkit for Multivariate Data Analysis, PoS **ACAT**, 040 (2007). arXiv: physics/0703039

[60] Buckley A (2015). LHAPDF6: parton density access in the LHC precision era. Eur. Phys. J. C.

[61] Gieseke S, Röhr C, Siódmok A (2012). Colour reconnections in Herwig++. Eur. Phys. J. C.

[62] ATLAS Collaboration, Studies on top-quark Monte Carlo modelling with Sherpa and MG5\_aMC@NLO, ATL-PHYS-PUB-2017-007 (2017). https://cds.cern.ch/record/2261938

[63] ATLAS Collaboration (2017). Search for triboson $$W^{\pm }W^{\pm }W^{\mp }$$ production in pp collisions at $$\sqrt{s} = 8\, TeV$$ with the ATLAS detector. Eur. Phys. J. C.

[64] ATLAS Collaboration (2018). Measurement of the production cross-section of a single top quark in association with a Z boson in proton-proton collisions at 13 TeV with the ATLAS detector. Phys. Lett. B.

[65] ATLAS Collaboration, Electron and photon energy calibration with the ATLAS detector using data collected in 2015 at $$\sqrt{s} = 13\, TeV$$, ATL-PHYS-PUB-2016-015, 2016. https://cds.cern.ch/record/2203514

[66] ATLAS Collaboration, Jet energy scale measurements and their systematic uncertainties in proton–proton collisions at $$\sqrt{s} = 13\, TeV$$ with the ATLAS detector, Phys. Rev. D **96** (2017) 072002, arXiv: 1703.09665 [hep-ex]

[67] ATLAS Collaboration, Luminosity determination in pp collisions at $$\sqrt{s} = 8\, TeV$$ using the ATLAS detector at the LHC. Eur. Phys. J. C **76**, 653 (2016). arXiv:1608.03953 [hep-ex] 10.1140/epjc/s10052-016-4466-1 PMC5335615 28316496

[68] Cowan G, Cranmer K, Gross E, Vitells O (2011). Asymptotic formulae for likelihood-based tests of new physics. Eur. Phys. J. C.

[69] Read AL (2002). Presentation of search results: the $$CL_{s}$$ technique. J. Phys. G.

[70] ATLAS Collaboration, ATLAS Computing Acknowledgements, ATL-GEN-PUB-2016-002. https://cds.cern.ch/record/2202407

